# Urban Cholera Transmission Hotspots and Their Implications for Reactive Vaccination: Evidence from Bissau City, Guinea Bissau

**DOI:** 10.1371/journal.pntd.0001901

**Published:** 2012-11-08

**Authors:** Andrew S. Azman, Francisco J. Luquero, Amabelia Rodrigues, Pedro Pablo Palma, Rebecca F. Grais, Cunhate Na Banga, Bryan T. Grenfell, Justin Lessler

**Affiliations:** 1 Department of Epidemiology, Johns Hopkins Bloomberg School of Public Health, Baltimore, Maryland, United States of America; 2 Epicentre, Paris, France; 3 National Public Health, Ministry of Health, Bissau City, Guinea-Bissau; 4 Médicos Sin Fronteras, Barcelona, Spain; 5 Department of Ecology and Evolutionary Biology, Princeton University, Princeton, New Jersey, United States of America; 6 Fogarty International Center, National Institutes of Health, Bethesda, Maryland, United States of America; University of California San Diego School of Medicine, United States of America

## Abstract

**Background:**

Use of cholera vaccines in response to epidemics (reactive vaccination) may provide an effective supplement to traditional control measures. In Haiti, reactive vaccination was considered but, until recently, rejected in part due to limited global supply of vaccine. Using Bissau City, Guinea-Bissau as a case study, we explore neighborhood-level transmission dynamics to understand if, with limited vaccine and likely delays, reactive vaccination can significantly change the course of a cholera epidemic.

**Methods and Findings:**

We fit a spatially explicit meta-population model of cholera transmission within Bissau City to data from 7,551 suspected cholera cases from a 2008 epidemic. We estimated the effect reactive vaccination campaigns would have had on the epidemic under different levels of vaccine coverage and campaign start dates. We compared highly focused and diffuse strategies for distributing vaccine throughout the city. We found wide variation in the efficiency of cholera transmission both within and between areas of the city. “Hotspots”, where transmission was most efficient, appear to drive the epidemic. In particular one area, Bandim, was a necessary driver of the 2008 epidemic in Bissau City. If vaccine supply were limited but could have been distributed within the first 80 days of the epidemic, targeting vaccination at Bandim would have averted the most cases both within this area and throughout the city. Regardless of the distribution strategy used, timely distribution of vaccine in response to an ongoing cholera epidemic can prevent cases and save lives.

**Conclusions:**

Reactive vaccination can be a useful tool for controlling cholera epidemics, especially in urban areas like Bissau City. Particular neighborhoods may be responsible for driving a city's cholera epidemic; timely and targeted reactive vaccination at such neighborhoods may be the most effective way to prevent cholera cases both within that neighborhood and throughout the city.

## Author Summary

Cholera remains a major public health threat, causing 3–5 million cases and 100,000–120,000 deaths each year. In 2010, data on vaccine performance and the changing epidemiology of cholera prompted the WHO's Strategic Advisory Group to recommend that reactive vaccination be considered in specific areas. We built a spatially explicit stochastic model of cholera transmission and fit it to data from a 2008 epidemic in Bissau City, Guinea Bissau. Using this model we examined the potential effectiveness of reactive vaccination for controlling cholera transmission in Bissau City, comparing strategies for distributing limited vaccine. In simulations, early targeting of a single transmission “hotspot”, Bandim, was the most effective strategy, and led to the greatest reduction in cases both within Bandim and in areas where no vaccine was distributed. This finding has implications for cholera control in urban settings in general: public health officials will often know which areas of a city were hotspots of cholera transmission in the past or where conditions promote efficient transmission. When there is limited vaccine, our work suggests that targeting reactive vaccination at these areas will lead to the greatest reduction in cases both in these areas and elsewhere in the city.

## Introduction

With the introduction of inexpensive, easy to administer, and effective oral vaccines against cholera, vaccination in response to an epidemic (reactive vaccination) may be an effective supplement to conventional control measures. Two safe and internationally licensed oral cholera vaccines are currently available, Dukoral and Shanchol. Both protect against clinical cholera two or more years after vaccination, but neither confers long lasting immunity [1]–[4]. On an epidemic timescale, these vaccines have efficacies ranging from 66 to 86% [2], [5].

Vaccination against cholera has been used preventatively [3], [6]–[8], but before 2012, we know of only two instances, in The Federated States of Micronesia in 2000 and Vietnam in 2008, where vaccination commenced during an epidemic [4], [9]. Vaccine efficacy estimates ranged from 76 to 80%, however, no analysis on how vaccination affected the course of the epidemic was reported for either case [4], [9].

New data on vaccine performance and the changing epidemiology of cholera prompted the WHO's Strategic Advisory Group to recommend in 2010 that reactive vaccination be considered in specific areas [10]. In order to facilitate rapid procurement and deployment of an oral cholera vaccine, some have proposed the creation of a revolving global stockpile [11], [12]. While discussions of the global stockpile proceed, countries that use reactive vaccination must contend with a limited supply that may arrive after a significant delay.

Spatial heterogeneities may influence how cholera vaccine can best be distributed in a reactive campaign. The effectiveness of a campaign and optimal allocation strategy will depend upon local cholera transmission dynamics, vaccine supply, and logistical delays [12], [13]. Human movement, water and sewerage infrastructure, and natural waterways facilitate cholera transmission across a city. Within neighborhoods, there can be marked variation in the efficiency of transmission.

One country that may benefit from reactive vaccination is Guinea-Bissau, where outbreaks have occurred every three to four years since 1994. Sector Autónomo de Bissau (SAB), or Bissau City, the capital, consistently reports the most cholera cases within the country (unpublished data, Guinea-Bissau Ministry of Health). In 2008, 67% of reported cases occurred in SAB while only 25% of the national population live within its boundaries [14]. Reactive vaccination in SAB may be possible in future epidemics given the concentration of cases within the city and the Ministry of Health's experience with vaccination campaigns.

Here, we explore the possible effectiveness of different reactive vaccination strategies using SAB as a case study. We fit a neighborhood-based meta-population model to the 2008 cholera epidemic. Using this model, we characterize the spatio-temporal dynamics of cholera transmission within the city and estimate the impact that different reactive vaccination strategies could have had on the course of the epidemic.

## Methods

### Data Sources

**Figure 1:**
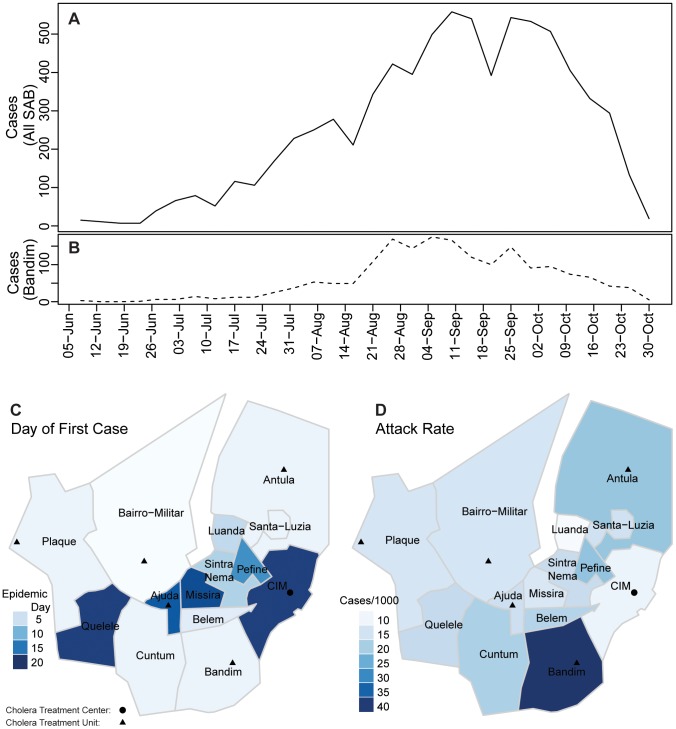
The 2008 cholera epidemic in SAB. Panel A (solid line) shows suspected and confirmed cholera cases reporting to cholera treatment centers/units (shown as circles and triangles) throughout all areas of SAB aggregated in 5-day intervals. The dashed line below (B) shows 5-day aggregated cases from Bandim, the area with the highest attack rate (40.6 per 1000). Panel C illustrates the day of the first reported case for each area. Attack rates (per 1000) for each area are shown in D.

During the 2008 epidemic, the Guinea-Bissau Ministry of Health, the WHO, and Mèdecins Sans Frontières implemented a clinic-based cholera surveillance system, which has been described previously [15]. In brief, upon arrival at either the cholera treatment center in the Hospital National Simao Mendes or one of five cholera treatment units (Figure 1C and 1D), health care providers entered patients into a surveillance registry. A patient's age, sex, area of residence, treatment facility, date of presentation, and clinical diagnosis were recorded.

Modified WHO cholera case definitions were used [15]. A suspected case was any person suffering from acute watery diarrhea, and a confirmed case was a suspected case with a positive stool sample containing *Vibrio cholerae* O1 or O139. We included all suspected and confirmed cases with complete information on their presentation date and home sanitary area in this analysis. The population for each sanitary area within the city was extrapolated from 1991 census data using a constant linear growth rate estimated by the Direcção-Geral Saúde. To estimate the population density in each sanitary area we traced the residential areas using Google Earth (v6.0.3.2197), then divided each sanitary area's population by its estimated residential area.

### Model of Cholera Spread in SAB

We fit a discrete-time Susceptible-Infectious-Recovered meta-population model to the confirmed and suspected cases reported during the 2008 epidemic with each of 14 sanitary areas in SAB treated as a distinct population. We assume the epidemic follows a first-order Markov process with a fixed generation time of five days. At each time step, the incidence in each area follows a Poisson distribution with a mean determined by the number infected in the last time step in all areas and the proportion of the area's population remaining susceptible. After infection, individuals were assumed to remain immune for the duration of the epidemic (See Text S1 for model details).

We considered models of cholera transmission with and without seasonality assuming (A) equal transmission coefficients between and within all areas of SAB; (B) different transmission coefficients within each area and equal transmission coefficients between all areas; (C) different transmission coefficients within each area and unique symmetric transmission coefficients between each pair of areas; and, (D) different transmission coefficients within each area and unique asymmetric transmission coefficients between each pair of areas in the city. We chose the best model based on Deviance Information Criteria (Text S1). To assess fit we simulated 300,000 epidemics predicting five, fifteen, and fifty days ahead drawing new parameters from the posterior distribution every 1000 simulations.

Posterior distributions were approximated using Markov Chain Monte Carlo methods using JAGS 3.1.0 and R 2.14.0 with non-informative priors [16], [17]. We ran 3 chains of 400,000 iterations with a burn in of 50,000, and assessed convergence using the potential scale reduction factor and through visual inspection [18].

### Vaccination

We assume every vaccinated individual receives two doses in a vaccine campaign over a 20 day period and that 75% are fully protected (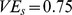 [19]) [3], [5], [6], [20]. In our model vaccinees get no protection until 10 days after the second dose [21], [22]. Hence, 75% of the susceptible vaccinees are considered immune starting 30 days after their first dose, with no protection before (Table 1).

**Table 1:** Overview of assumptions related to vaccination and immunity.

Vaccine efficacy	75%
Doses per individual	2
Immunity before second vaccine dose	None
Duration of vaccination campaign	20 days
Time from second vaccine dose to complete protection	10 days
Proportion immune after natural infection	100%
Length of immunity from natural infection or successful vaccination	Duration of the epidemic

Main assumptions used in primary analysis related to vaccination and immunity. Additional details are provided in the methods section and Text S1.

We considered campaigns with 50,000, 75,000, or 100,000 doses (i.e. 25,000, 37,500, and 50,000 individuals vaccinated) and targeted vaccination at one, two, three, or all (14) areas (Table 3). When the proposed number of vaccinees in a specific area exceeded the population size, we distributed vaccine to the other vaccination areas or, in the campaigns with one vaccination area, we dispersed the vaccine throughout the city with each person having equal probability of getting vaccinated. We varied the starting time of the vaccination campaign between 20 and 120 days after the first case was detected.

We considered targeted and diffuse (city-wide) campaigns. In diffuse campaigns, vaccine was distributed throughout all areas of SAB. In targeted campaigns, we considered three different strategies to select vaccination areas. In the population-based strategy, we selected the areas with the largest population. In the connectivity-based strategy, we vaccinated in areas estimated to be most “connected” to other areas. In the attack rate-based strategy, we chose the areas with the highest attack rate in the 2008 epidemic. We allocated vaccine proportional to population size in all simulations.

### Simulation Studies

For each vaccination scenario we ran 5,000 simulations calculating the difference between the final epidemic size with and without vaccination. Epidemics were assumed to follow the observed 2008 epidemic course until 30 days after the first dose. In each simulation we drew new parameters from the joint posterior distribution. As a sensitivity analysis, we ran simulations with different generation times (3–10 days) and vaccine efficacies (65%–85%). Additional simulation study details are available in Text S1.

### Ethics Statement

Original data collection was approved by the Mèdecins Sans Frontières ERB and the National Ethical Review Board of Guinea-Bissau [15]. The analyses presented in this article were conducted on de-identified data and deemed to be non-human subject research by the Johns Hopkins Bloomberg School of Public Health IRB.

## Results

### The 2008 Cholera Epidemic

The first case in SAB was reported on June 5, 2008 in Bairro-Militar, the most populated area of the city (Figures 1A, 1B), one month after the first reported case in Guinea-Bissau. Within three weeks, all 14 areas had reported cases (Figure 1C). The Ministry of Health officially declared an epidemic one month after the first case report from SAB. The National Laboratory of Microbiology and the Pasteur Laboratory in Dakar, Senegal identified all positive specimens analyzed as *Vibrio cholerae* O1 El Tor Ogawa.

Nationally, 14,226 suspected cases and 228 deaths were reported with 67% (9,393) of cases and 32% (73) of deaths reported in SAB. The last case in the country was reported in SAB on January 11, 2009. Individual-level data in SAB was collected between June 5, 2008 and October 28, 2008, over which time 8,024 (85%) suspected and confirmed cases were reported. These analyses focus on 7,551 suspected and confirmed cases with complete information on date of presentation, home area, and clinical diagnosis (Figure S1).

In SAB, weekly incidence ranged from 14 to 755. Within-area attack rates ranged from 9.1 to 40.6 per 1,000 (Table 2, Figure 1D), with Bandim having both the most cases (1,816) and the highest attack rate.

**Table 2:** Overview of sanitary areas in SAB.

Sanitary Area	Population	Suspected and Confirmed Cases	Attack Rate (per 1,000)
Barrio-Militar	65,274	944	14.5
Bandim	44,718	1,816	40.6
Cuntum	45,482	890	19.6
Missira	38,838	532	13.7
Antula	30,778	662	21.5
Quelele	28,898	493	17.1
Plaque	27,633	396	14.3
Luanda	25,236	229	9.1
Sintra Nema	21,451	355	16.5
Belem	17,263	322	18.7
Santa-Luzia	17,204	261	15.2
CIM	14,985	161	10.7
Pefine	14,808	324	21.9
Ajuda	10,429	164	15.7
All SAB	402,997	7,549	18.7

Estimated 2008 population for each sanitary area projected from 1991 census data (second column). Suspected and confirmed cases with complete location and time data and attack rate during 2008 cholera epidemic (third and fourth columns).

**Table 3:** Vaccination scenarios.

	Vaccination Strategy				
Areas Vaccinated	Population	Connectivity	Attack Rate	Vaccination Start Day	Doses
1 Area	Bairro Militar (1.00)	Missira (1.00)	Bandim (1.00)	20–120	50,000–100,000
2 Areas	Bairro Militar (0.59)	Missira (0.69)	Bandim (0.75)	20–120	50,000–100,000
	Cuntum (0.41)	Santa-Luzia (0.31)	Pefine (0.25)		
3 Areas	Bairro Militar (0.42)	Missira (0.46)	Bandim (0.50)	20–120	50,000–100,000
	Cuntum (0.29)	Santa-Luzia (0.21)	Pefine (0.16)		
	Bandim (0.29)	Plaque (0.33)	Antula (0.34)		

For each scenario we chose the top 1, 2, and 3 areas that met the vaccination strategy criteria. The number of vaccinees in each area were weighted (shown in parenthesis) to ensure that vaccines were allocated proportional to population size in all simulations.

### Spatial Spread of Cholera in SAB

**Figure 2:**
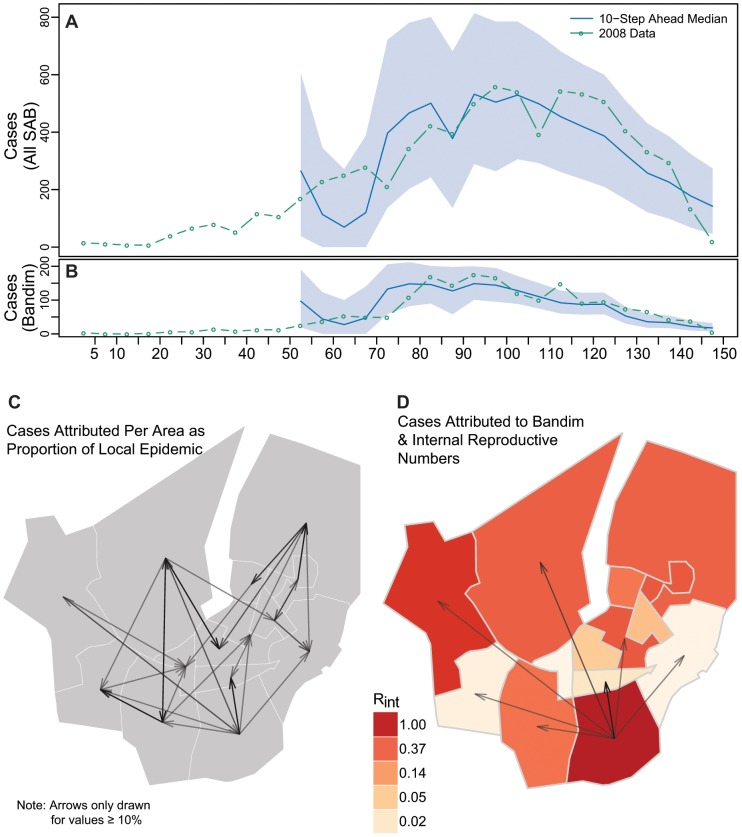
Cholera transmission model overview. 10-step ahead (50 day) predictions for all of SAB (A) and Bandim (B) with 95% predictive interval bands. The arrows in Panel C illustrate the proportion of cases estimated to be caused in each area (head of arrow) by another (tail end of arrow). Panel D illustrates the mean effective internal reproductive number (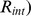 for each area (colors), and the proportion of each areas epidemic estimated to be caused by Bandim (arrows). Arrow size and transparency are scaled by the magnitude with a minimum of 10% shown.

**Figure 3:**
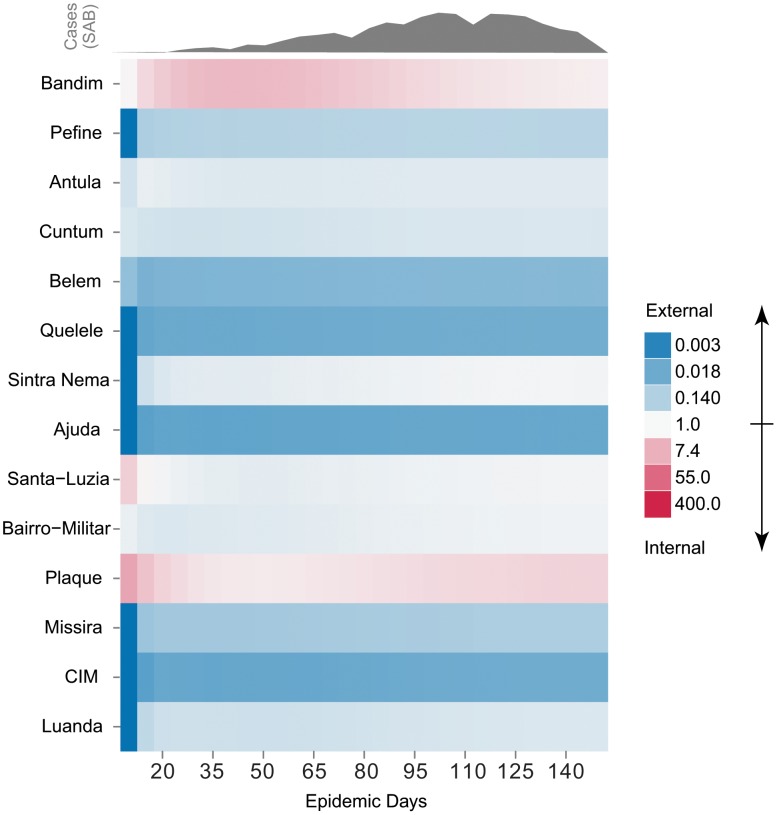
Odds of internally caused case over time by area. Odds of a case being caused internally (i.e. as a result of other cases in that area) vs. externally for all areas throughout the epidemic, sorted by attack rate (top to bottom). Red represents those values in support of an internally driven epidemic and blue represents those supporting an externally driven epidemic. The observed epidemic curve is shown above in grey for reference.

The final model fit both the overall and area-specific epidemic curves well, even when predicting as far as 50 days (i.e. 10 time steps) ahead (Figures 2A,2B). To understand how transmission varied through time, we calculated the odds that an incident case was caused locally (i.e. attributable to transmission between people in the same area) for each area throughout the course of the epidemic (Figure 3). Only Bandim, Plaque, and Santa-Luzia have an odds consistently greater than 1, suggesting internally driven epidemics in these areas.

**Figure 4:**
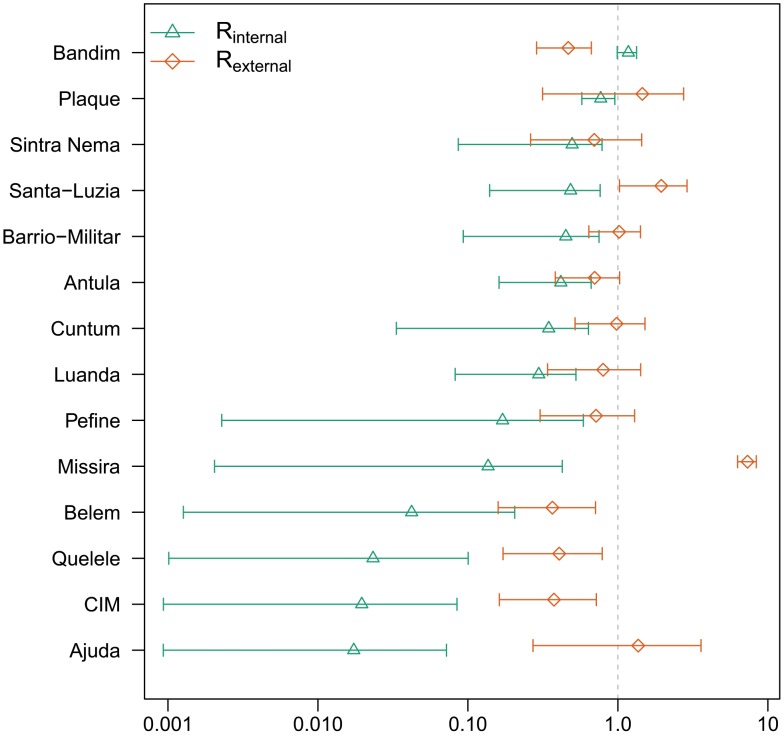
Mean 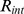, 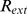 and 95% credible intervals. Sorted from top to bottom by 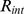.

We define the effective internal basic reproductive number (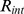) as the expected number of cases caused within a given area by one infected individual, within the same area, at the beginning of the epidemic. Only areas with 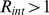 can sustain an epidemic absent infections introduced from other areas. The strength of internal epidemics varied with estimates of 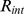 ranging from 0.01 (95% Credible Interval (CI) 0.00–0.07) in Ajuda to 1.17 (95% CI 0.99–1.33) in Bandim (Figure 4). We found no significant correlation between 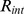 and either estimated population size or population density.

Bandim is the only area where we estimate 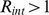, and it appears to have played a necessary role in driving the epidemic. With Bandim removed, simulated introductions of cases fail to cause epidemics. In contrast, city-wide epidemics occur with removal of any other single area.

In simulated epidemics based upon our best-fit model, we find that, on average, at least 10% of cases in each area are caused by cases in other areas (Figure 2C, Text S1). External transmission coefficients represent epidemic connectivity between areas, and our estimates suggest heterogeneity in inter-area transmission (Text S1). Based on simulations, we estimate that Bandim contributed over 10% of the cases to over half (7/13) of the other areas (Figure 2D), highlighting the crucial role it played in the epidemic.

The sum of the external transmission coefficients for any area provides an estimate of the effective external basic reproductive number (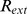). This number is the estimated number of cases a single infectious case in that area would cause in all other areas of SAB given the pre-epidemic level of population immunity. Estimates of 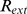 ranged from 0.37 (95% CI 0.16–0.71) in Belem to 7.32 (95% CI 6.29–8.37) in Missira (Figure 4).

### Reactive Vaccination Simulations

**Figure 5:**
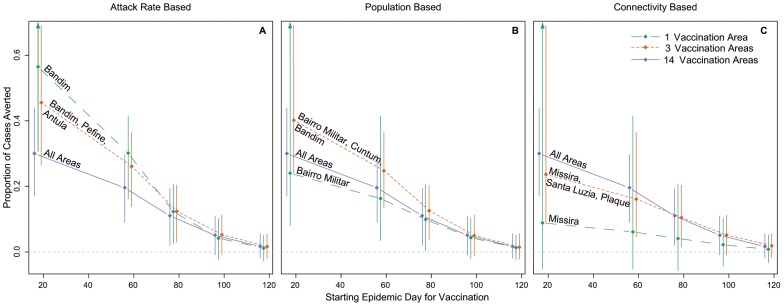
Vaccination results by strategy and start time. Each plot shows the median (diamonds) and 95% predictive interval for the proportion of cases averted by vaccination start time for (A) attack rate-based, (B) population-based, and (C) connectivity-based targeting strategies. The colored lines represent the different number of areas vaccinated. Estimates made from simulations starting at the time of vaccination with 37,500 individuals vaccinated (75,000 doses). Purple lines (14 vaccination areas) are the same in each panel.

Vaccination in the area(s) with the highest attack rate leads to larger reduction in cases than all other targeted and city-wide campaigns at all starting times. Targeting vaccination at Bandim only, the area with the highest attack rate, within the first 80 days of the epidemic averts more cases than other strategies regardless of vaccine quantity (Figure 5). Targeted vaccination in Bandim starting on day 20 is expected to reduce the final size of the epidemic by 41% (95% Predictive Interval (PI) 0.21–0.69), 56% (95% PI 0.30–0.85), and 67% (95% PI 0.40–0.89) with 25,000, 37,500, and 50,000 vaccinees, respectively. In comparison, a city-wide campaign starting on the same day is expected to reduce the epidemic size by 21% (95% PI 0.07–0.34), 30% (95% PI 0.17–0.44), and 40% (95% PI 0.27–0.55) for 25,000, 37,500, and 50,000 vaccinees (Tables 4,S1,S2).

**Table 4:** Vaccination scenario results summary.

		Vaccination Campaign Start Time							
Distribution Strategy	# Areas Vacc.	Day 20		Day 60		Day 80		Day 100	
		Cases	%	Cases	%	Cases	%	Cases	%
**Attack Rate**	1 area	4228	0.56	2342	0.30	970	0.12	345	0.04
		2263,6424	0.30,0.85	1195,3392	0.16,0.41	197,1732	0.03,0.21	−186,887	−0.02,0.10
	2 areas	3954	0.53	2266	0.29	986	0.13	379	0.05
		2142,6214	0.29,0.82	1156,3258	0.16,0.40	238,1732	0.03,0.21	−146,928	−0.02,0.11
	3 areas	3422	0.46	2025	0.26	975	0.12	433	0.05
		1903,5174	0.27,0.69	1021,2993	0.14,0.36	222,1708	0.03,0.20	−71,964	−0.01,0.11
Population	1 area	1804	0.24	1272	0.16	777	0.10	359	0.04
		558,3250	0.08,0.41	254,2276	0.03,0.28	27,1565	0,0.19	−166,897	−0.02,0.10
	2 areas	1974	0.26	1405	0.18	859	0.11	396	0.05
		824,3355	0.12,0.42	432,2361	0.06,0.29	102,1633	0.01,0.19	−120,936	−0.02,0.11
	3 areas	3019	0.40	1928	0.25	996	0.13	414	0.05
		1727,4534	0.24,0.59	976,2902	0.13,0.35	269,1739	0.04,0.21	−92,941	−0.01,0.11
Connectivity	1 area	666	0.09	476	0.06	322	0.04	181	0.02
		−363,1742	−0.05,0.22	−404,1372	−0.05,0.17	−436,1102	−0.06,0.13	−349,716	−0.04,0.08
	2 areas	1258	0.17	827	0.11	566	0.07	326	0.04
		154,2375	0.02,0.3	−62,1741	−0.01,0.21	−129,1322	−0.02,0.16	−198,863	−0.03,0.10
	3 areas	1792	0.24	1255	0.16	828	0.10	427	0.05
		603,3032	0.09,0.39	339,2243	0.05,0.27	104,1574	0.01,0.19	−74,967	−0.01,0.11
**Diffuse/City-Wide**	14 areas	2271	0.30	1521	0.20	872	0.11	421	0.05
		1170,3450	0.17,0.44	658,2464	0.09,0.30	150,1623	0.02,0.19	−71,947	−0.01,0.11

Median count and percent of cases averted by targeting strategy (indicated by left-most column) and vaccination start day (epidemic day) for 75,000 doses (37,500 vaccinees). Values were estimated from simulations starting from the first time period where any vaccinee gained protective immunity. 95% predictive intervals (PIs) are shown below each median value. Differences were calculated from time that the first vaccinated individuals are protected.

We found wide variability in the outcomes using different targeting strategies, with the differences diminishing as vaccination is delayed (Figure 5). Under the population-based strategy, only a targeted campaign in the three most populated areas averts more cases than a city-wide campaign (Figure 5, Table 4). Targeting the areas estimated to be most “connected” to others averts fewer cases than city-wide campaigns regardless of vaccination starting time and doses.

Starting day has a profound impact on the effect of all vaccination campaigns: the sooner vaccination begins, the more cases are averted. With 37,500 vaccinees, each day delay in vaccination results in an average of 39.5 (95% CI 37.7–44.2) fewer cases averted when targeting based on attack rate. Increasing the size of a vaccination campaign early on in the epidemic can significantly improve case prevention, however, the marginal benefit of additional vaccine diminishes as vaccination is delayed. On average, each additional person vaccinated as part of a targeted campaign in Bandim starting on day 20 averts 7.5 cases compared to 1.7 cases averted per vaccinee in campaigns starting two months later.

**Figure 6:**
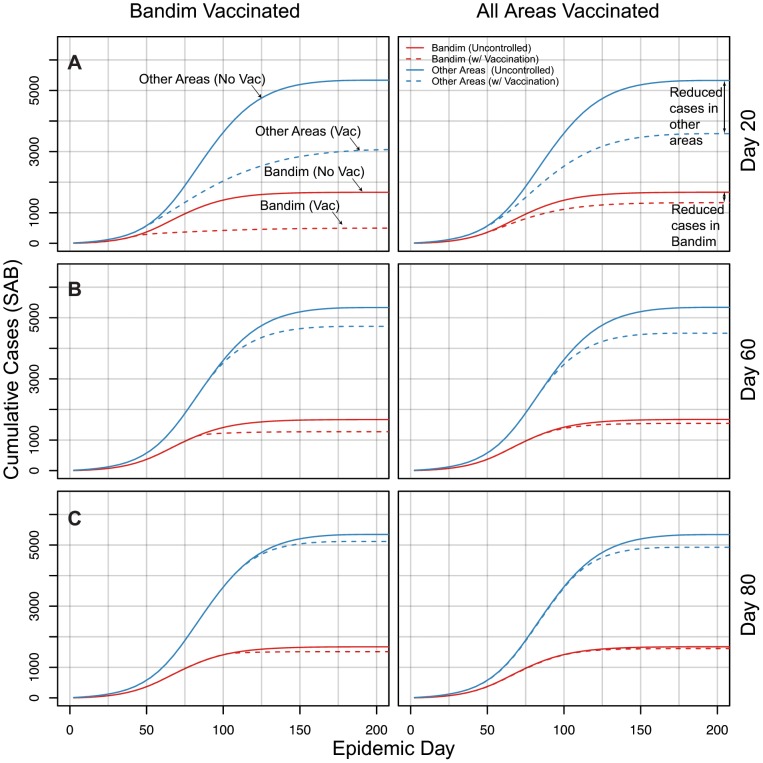
Comparison of cumulative cases within (red) and outside (blue) Bandim under targeted and diffuse vaccination. Dashed lines represent the median number of cases in simulations with vaccination, and the solid lines represent the median number of cases in uncontrolled epidemic simulations (no vaccination). Each row (panels A–C) represents simulations with vaccination started at the epidemic day denoted on the right hand side (e.g. Day 20). Simulations were started from the reported number of cases in the first 5 days of the epidemic.

In simulations, early targeted vaccination leads to fewer cases both within the targeted area *and throughout the city* when compared to diffuse campaigns. When starting vaccination on day 20 (Figure 6A), targeting Bandim averts more cases both in Bandim (1,173) and in all the other areas combined (2,265) when compared to a city-wide campaign (341 averted in Bandim and 1,741 in all other areas). As the vaccination campaign is delayed, these differences shrink (Figure 6).

## Discussion

Using a simple spatially explicit model of cholera transmission, we captured the essential dynamics of the 2008 cholera epidemic in SAB, Guinea-Bissau. This model suggests that there was significant transmission between areas in SAB and that one area, Bandim, drove the epidemic. Our simulations show that early distribution of vaccine is the most important determinant of the number of cases prevented. For example, vaccinating 25,000 individuals in Bandim on epidemic day 20 would have averted more cases (3,109, 95% PI 1,475–5,198) than vaccinating 50,000 in the same area just 40 days later (2,732, 95% PI 1,630–3,738). Our simulations suggest that an early vaccination campaign targeted at Bandim alone would have outperformed distributing the same vaccine quantity throughout the city. Not only are more cases prevented overall, but more are prevented in areas outside of Bandim.

Our results suggest that rapid small-scale vaccination may be more effective than a delayed larger-scale vaccination campaign. For example, on average, each day delay results in an additional 39.5 cases when targeting 37,500 people in the areas with the highest attack rate. Applying the average case fatality ratio from the 2008 epidemic (1.58 per 100 cases [15]) we estimate that each week delay in vaccination would have resulted in an average of 4.4 cholera-related deaths.

Transmission hotspots for other infectious diseases have been exploited to devise novel prevention and control approaches [23], [24]. For example, targeted interventions in hotspots may be key to effective malaria control and elimination [24]. Similarly, cholera hotspots can serve as targets for both reactive and preventative interventions. Identification of hotspots during an epidemic may be challenging. In the case of SAB, Bandim is an area which has had high attack rates in previous epidemics and few improvements in water and sanitation infrastructure. Such historical information may be useful in targeting vaccination; however, more research on combining historical and real-time surveillance data is needed.

In our model, vaccination campaigns lasted 20 days, but in reality the duration will vary by the number of vaccinees targeted and the vaccine used. If Shanchol were used with the recommended inter-dose period of 14 days, the campaign would likely exceed 20 days. While this suggests that our results underestimate the speed by which Shanchol vaccination would occur, these differences would be offset by partial immunity conferred before a second dose [22].

As the time to distribute vaccine doses increases, we expect to avert fewer cases. However, there is some evidence that a single dose of oral cholera vaccine may be sufficient for reactive vaccination [22], [25]. If one dose is sufficient to elicit a strong protective response for the time-scale of an epidemic, more people could be vaccinated quickly.

Cholera's generation time is not well characterized and varies widely with the concentration of bacteria in the environment, its survival rate, and the route of transmission [26]–[28]. We ran analyses with alternate generation times of 3, 7, and 10 days and got the same qualitative results (Figures S3, S4, S5, S6, S7, S8 and Tables S3, S4, S5). We also found that varying the vaccine efficacy to 65% and 85% changed the number of cases averted, but preserved the relative performance of each strategy over time (Figure S2 and Tables S7,S6).

There are a number of limitations to this work. We focus on a single epidemic in Guinea-Bissau. A longer time series would provide insight into variability in transmission across epidemics. The data came from an intensified surveillance effort from both Mèdecins Sans Frontières and the Guinea-Bissau Ministry of Health, however suspected cases that presented after October 28, 2008 were only captured by the national surveillance system without details on timing and home sanitary area.

There are several possible alternative explanations for the elevated attack rate in Bandim. The cholera case definition used is not 100% specific, and some cholera cases may be false positives. People may be more likely to seek care if their neighbors do, hence clinic visits may cluster even if cholera does not. In addition, Bandim has been the location to several surveillance programs and public health interventions through the Bandim Health Project [29], perhaps leading to increased awareness. However, if these phenomena were consistent throughout the epidemic they would not lead to elevated estimates of the local transmission rate under our algorithm.

We found that how rapidly vaccine can be distributed during a cholera epidemic is the most important determinant of the effectiveness of a reactive vaccination program; and that a single area of SAB was an essential driver of the epidemic. Hence, early targeting of this area would have been the most effective way to reactively distribute vaccine. These results may apply to urban cholera epidemics more generally. It seems reasonable that cholera epidemics in other urban settings, particularly in Africa, may be disproportionally driven by specific parts of the city. If these hotspots can be identified, targeted reactive vaccination may be an effective way to prevent cases both within that area and throughout the city, especially when vaccine supply is limited. Regardless of the distribution strategy used, timely distribution of vaccine in response to an ongoing cholera epidemic can prevent cases and save lives.

## Supporting Information

Figure S1 **5-day aggregated case counts for all sanitary areas during the 2008 epidemic.** Data collected from cholera treatment center and cholera treatment units throughout the city from June 5, 2008 to October 28, 2008.
(TIF)

Figure S2 **Vaccine efficacy sensitivity analysis.**Comparison of proportion of epidemic averted with different 65%, 75% (as in main analysis), and 85% vaccine efficacy over different vaccination starting times. All scenarios shown use attack rate based targeting.
(TIF)

Figure S3
**Comparison of transmission parameters with different generation times.** Posterior means and standard deviation for transmission coefficients, (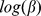
's on diagonals and 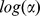
's on off-diagonals) with 3, 5, and 7 day generation times.(TIF)

Figure S4 **Comparison of internal and external effective reproductive numbers for different generation time aggregations.**
(TIF)

Figure S5 **Proportion of cases caused in each area by others from 3, 5, 7, and 10-day generation time models.** The sum of each row is equal to one, representing 100% of the area's epidemic.
(TIF)

Figure S6 **Vaccination simulation results with 3-day generation time, 75% vaccine efficacy, and 75,000 doses.**
(TIF)

Figure S7 **Vaccination simulation results with 7-day generation time, 75% vaccine efficacy, and 75,000 doses.**
(TIF)

Figure S8 **Vaccination simulation results with 10-day generation time, 75% vaccine efficacy, and 75,000 doses.**
(TIF)

Table S1 **Vaccination simulation results with 50,000 doses and 75% vaccine efficacy.** Proportion and number of cases averted in 5,000 simulations under different vaccination strategies (Median and 95% Predictive Interval).
(DOCX)

Table S2 **Vaccination simulation results with 100,000 doses and 75% vaccine efficacy.** Proportion and number of cases averted in 5,000 simulations under different vaccination strategies (Median and 95% Predictive Interval).
(DOCX)

Table S3 **Vaccination simulation results from 3-day generation time model, 75,000 doses.** Proportion and number of cases averted in 5,000 simulations under different vaccination strategies (Median and 95% Predictive Interval).
(DOCX)

Table S4 **Vaccination simulation results from 7-day generation time model, 75,000 doses.** Proportion and number of cases averted in 5,000 simulations under different vaccination strategies (Median and 95% Predictive Interval).
(DOCX)

Table S5 **Vaccination simulation results from 10-day generation time model.** Proportion and number of cases averted in 5,000 simulations under different vaccination strategies (Median and 95% Predictive Interval).
(DOCX)

Table S6 **Vaccination simulation results with 75,000 doses and 65% vaccine efficacy.** Proportion and number of cases averted in 5,000 simulations under different vaccination strategies (Median and 95% Predictive Interval).
(DOCX)

Table S7 **Vaccination simulation results with 75,000 doses and 85% vaccine efficacy.** Proportion and number of cases averted in 5,000 simulations under different vaccination strategies (Median and 95% Predictive Interval).
(DOCX)

Text S1 **Details on final model, model selection, and simulations.**
(PDF)
