# Supplementary figures and images for: Urban Cholera Transmission Hotspots and Their Implications for Reactive Vaccination: Evidence from Bissau City, Guinea Bissau
Source: PLoS Negl Trop Dis. 2012 Nov 8;6(11):e1901. doi: 10.1371/journal.pntd.0001901 (PMC3493445; doi:10.1371/journal.pntd.0001901)

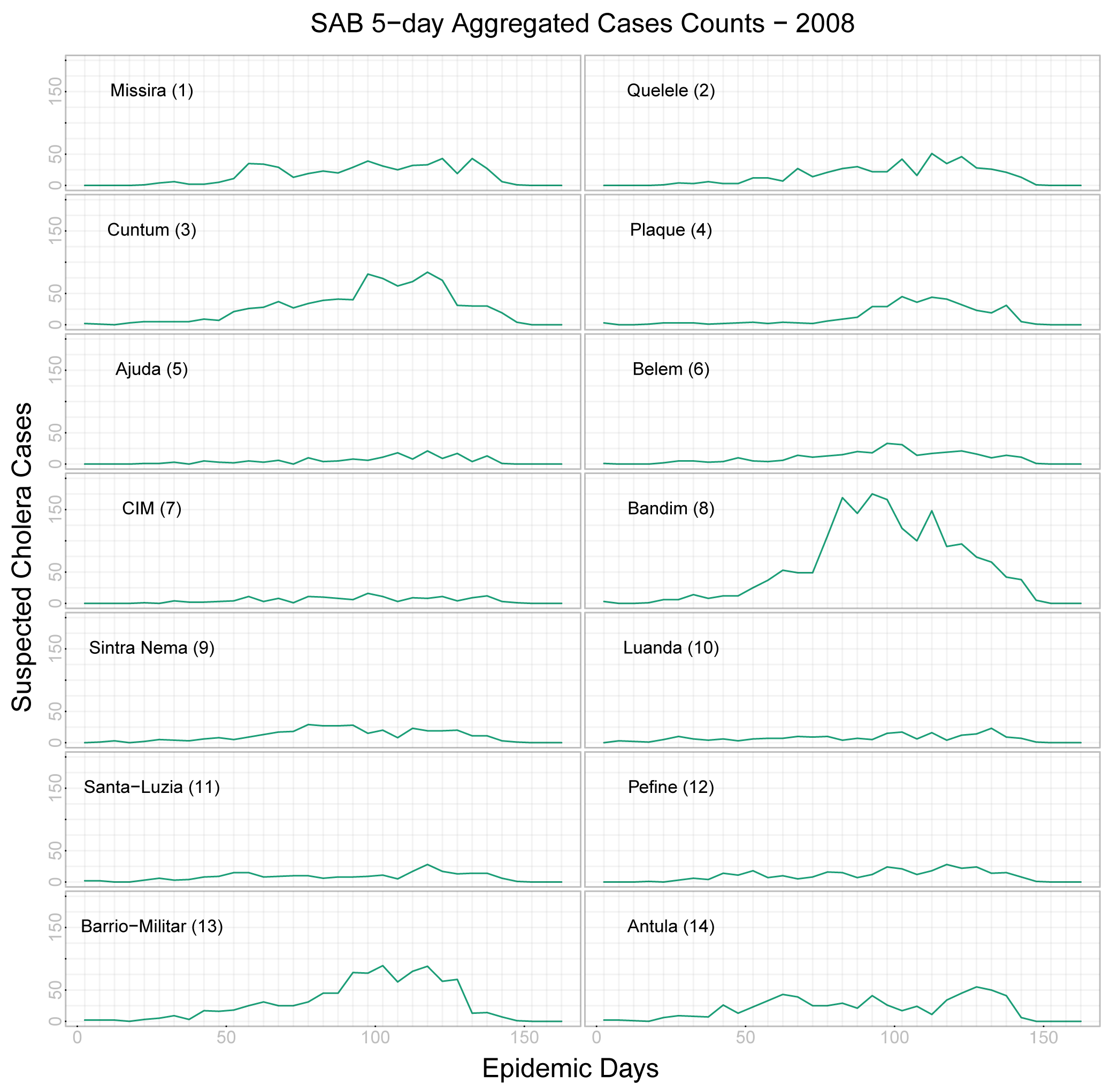

Supplement: Figure S1 — 5-day aggregated case counts for all sanitary areas during the 2008 epidemic. Data collected from cholera treatment center and cholera treatment units throughout the city from June 5, 2008 to October 28, 2008. (TIF) [file pntd.0001901.s001.tif]

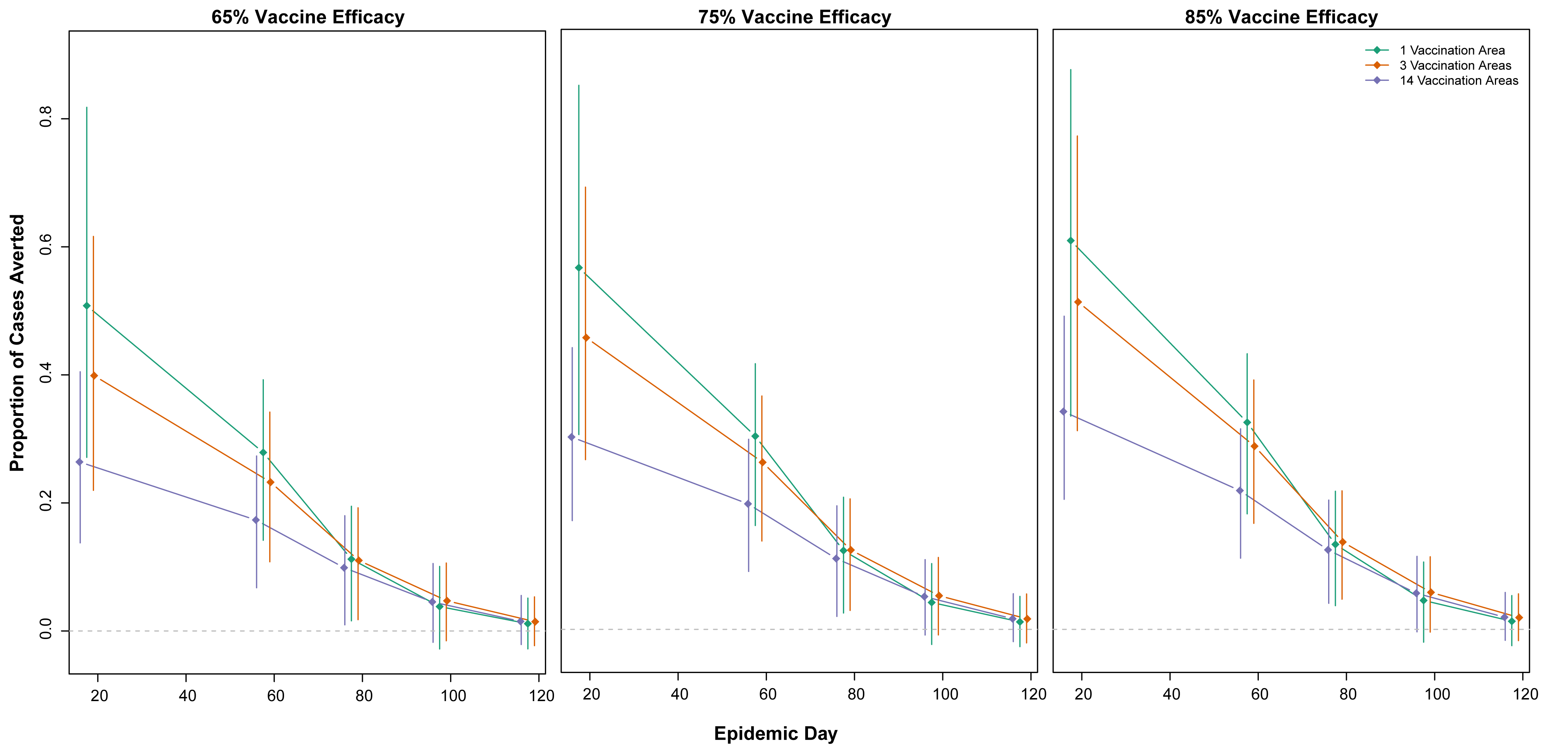

Supplement: Figure S2 — Vaccine efficacy sensitivity analysis.Comparison of proportion of epidemic averted with different 65%, 75% (as in main analysis), and 85% vaccine efficacy over different vaccination starting times. All scenarios shown use attack rate based targeting. (TIF) [file pntd.0001901.s002.tif]

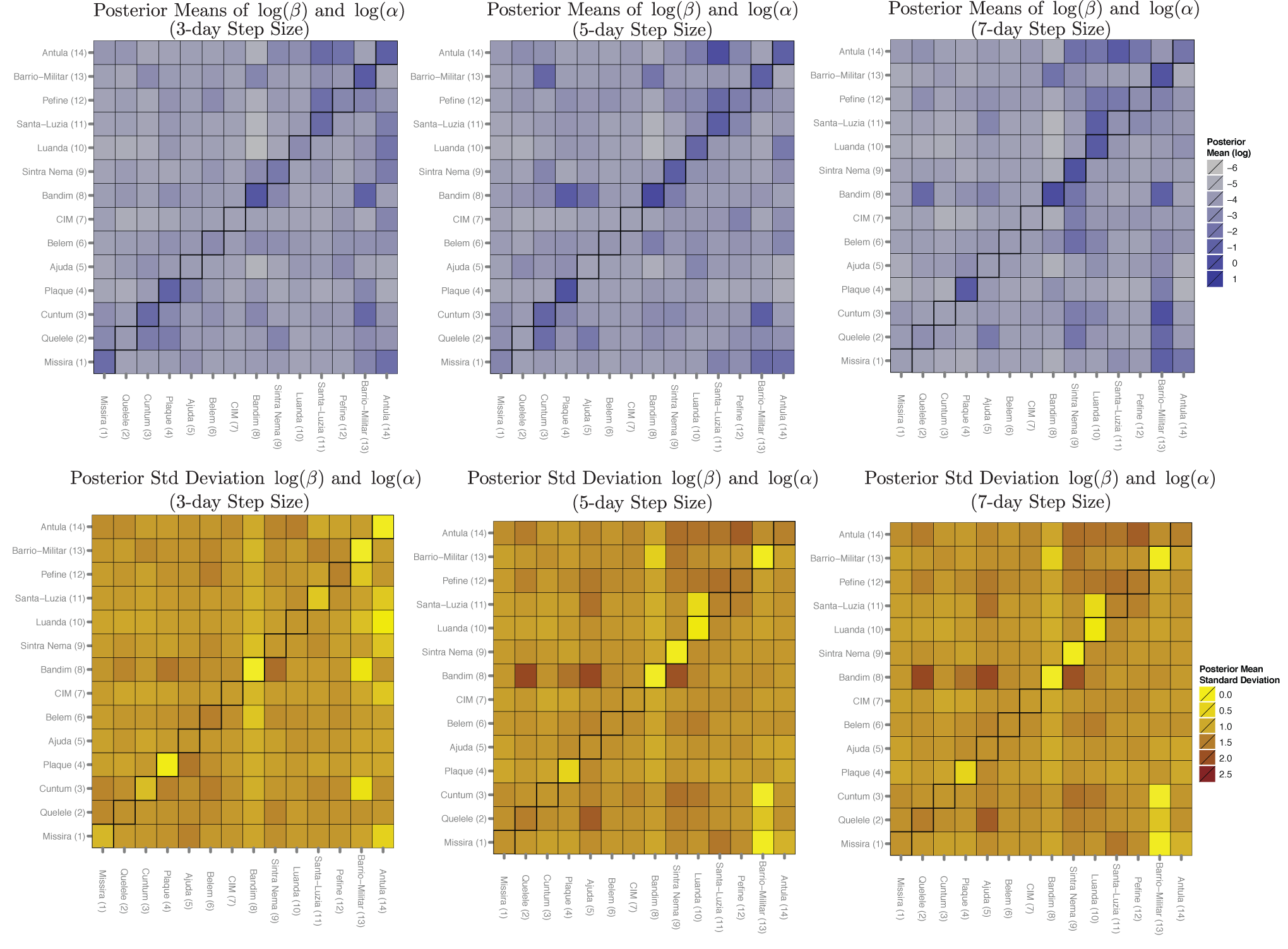

Supplement: Figure S3 — Comparison of transmission parameters with different generation times. Posterior means and standard deviation for transmission coefficients, ('s on diagonals and 's on off-diagonals) with 3, 5, and 7 day generation times. (TIF) [file pntd.0001901.s003.tif]

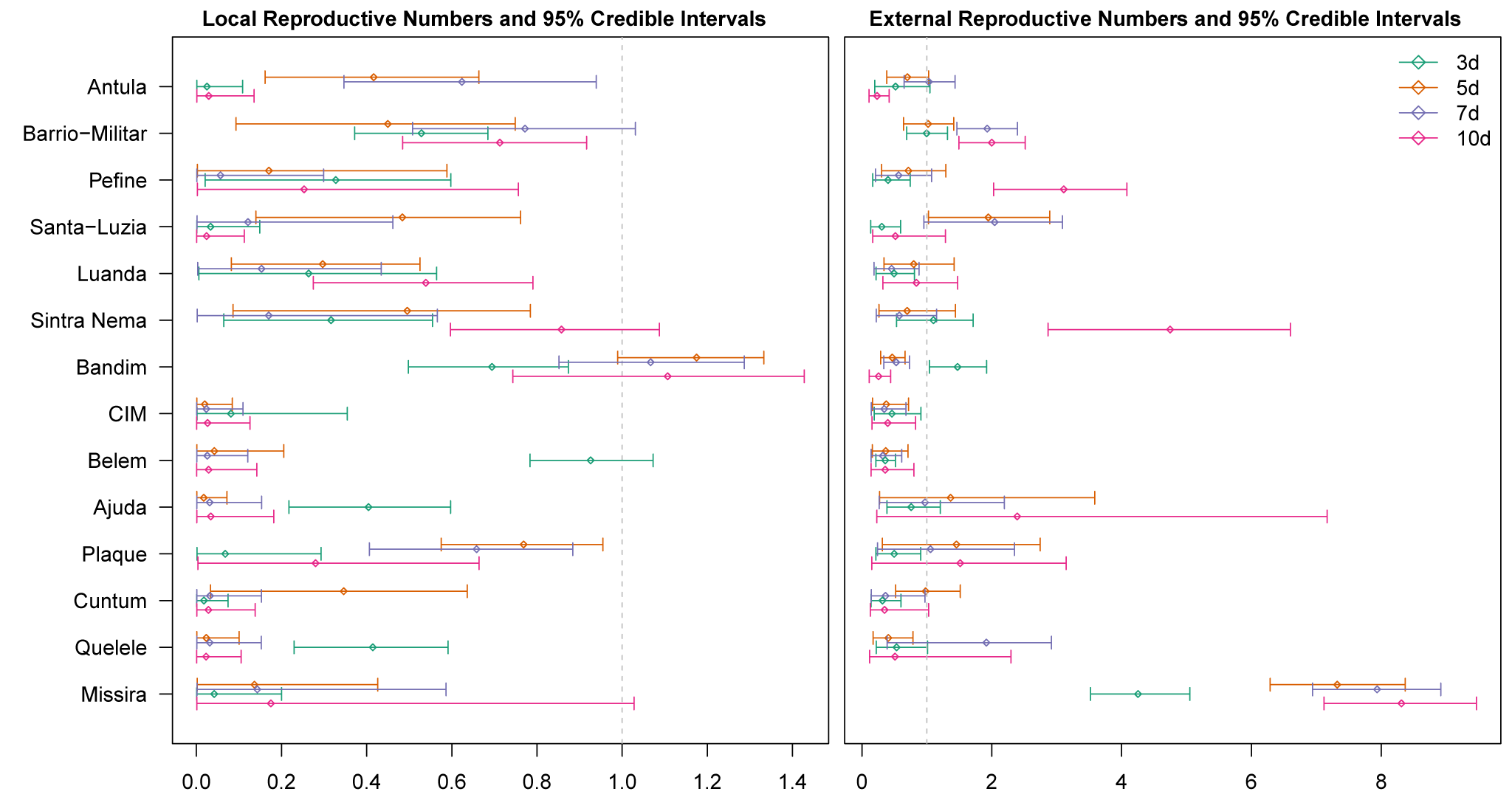

Supplement: Figure S4 — Comparison of internal and external effective reproductive numbers for different generation time aggregations. (TIF) [file pntd.0001901.s004.tif]

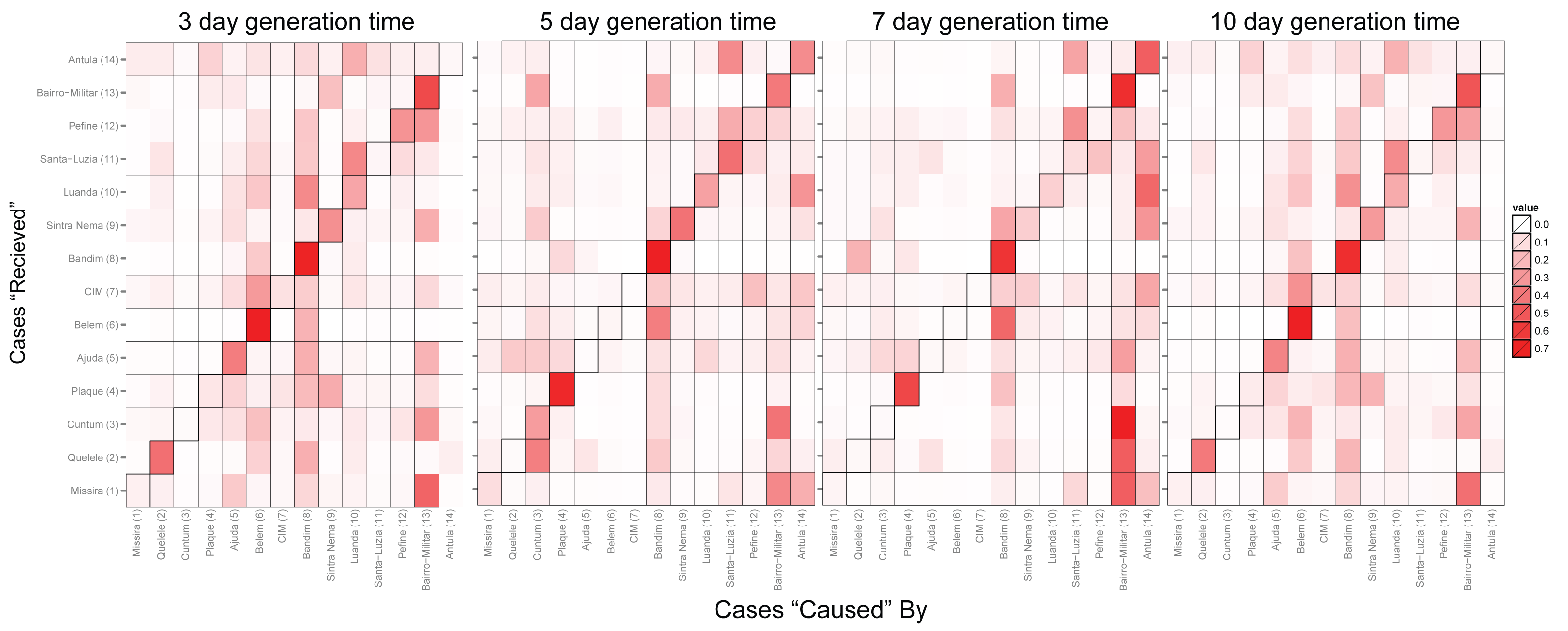

Supplement: Figure S5 — Proportion of cases caused in each area by others from 3, 5, 7, and 10-day generation time models. The sum of each row is equal to one, representing 100% of the area's epidemic. (TIF) [file pntd.0001901.s005.tif]

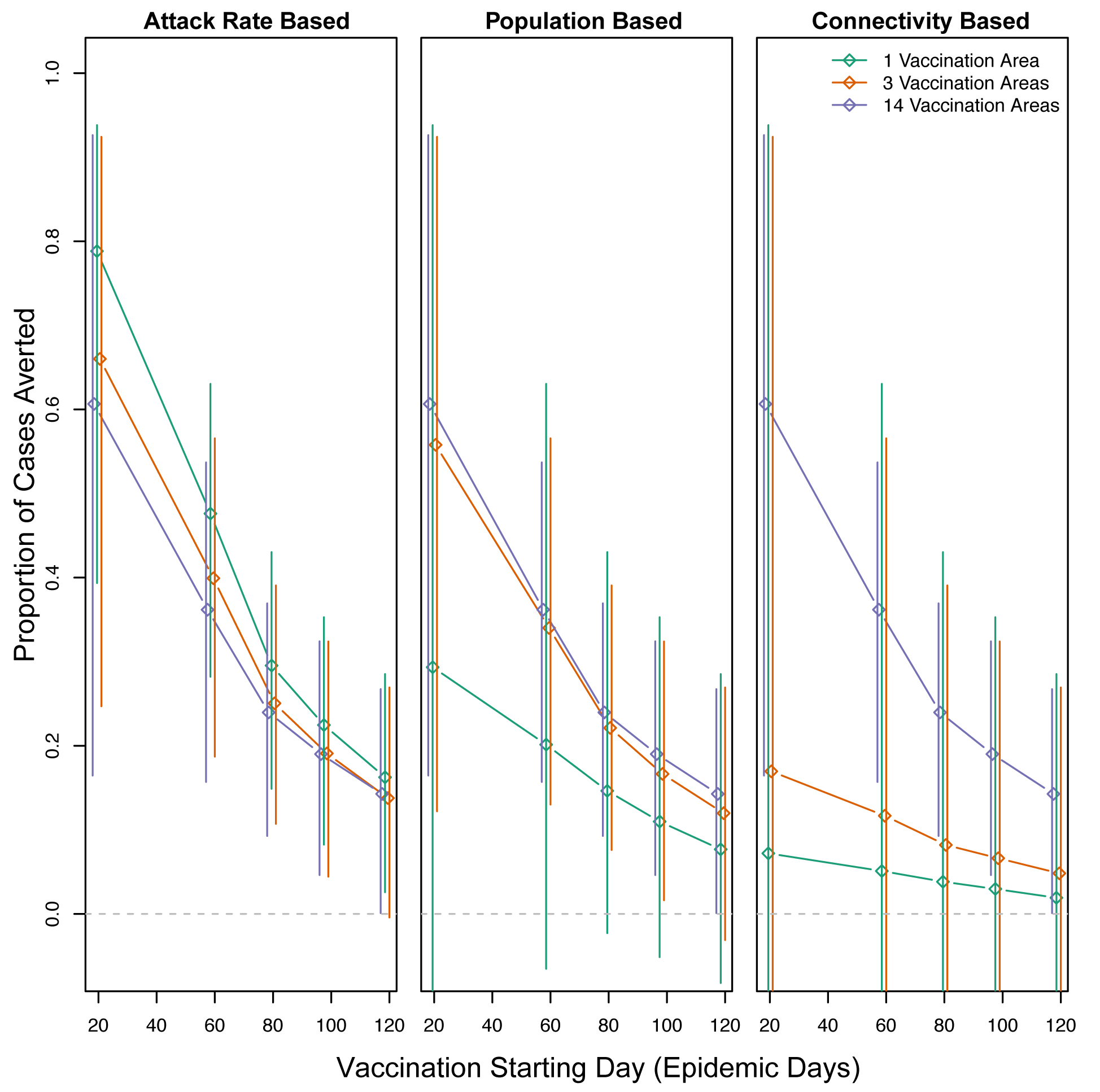

Supplement: Figure S6 — Vaccination simulation results with 3-day generation time, 75% vaccine efficacy, and 75,000 doses. (TIF) [file pntd.0001901.s006.tif]

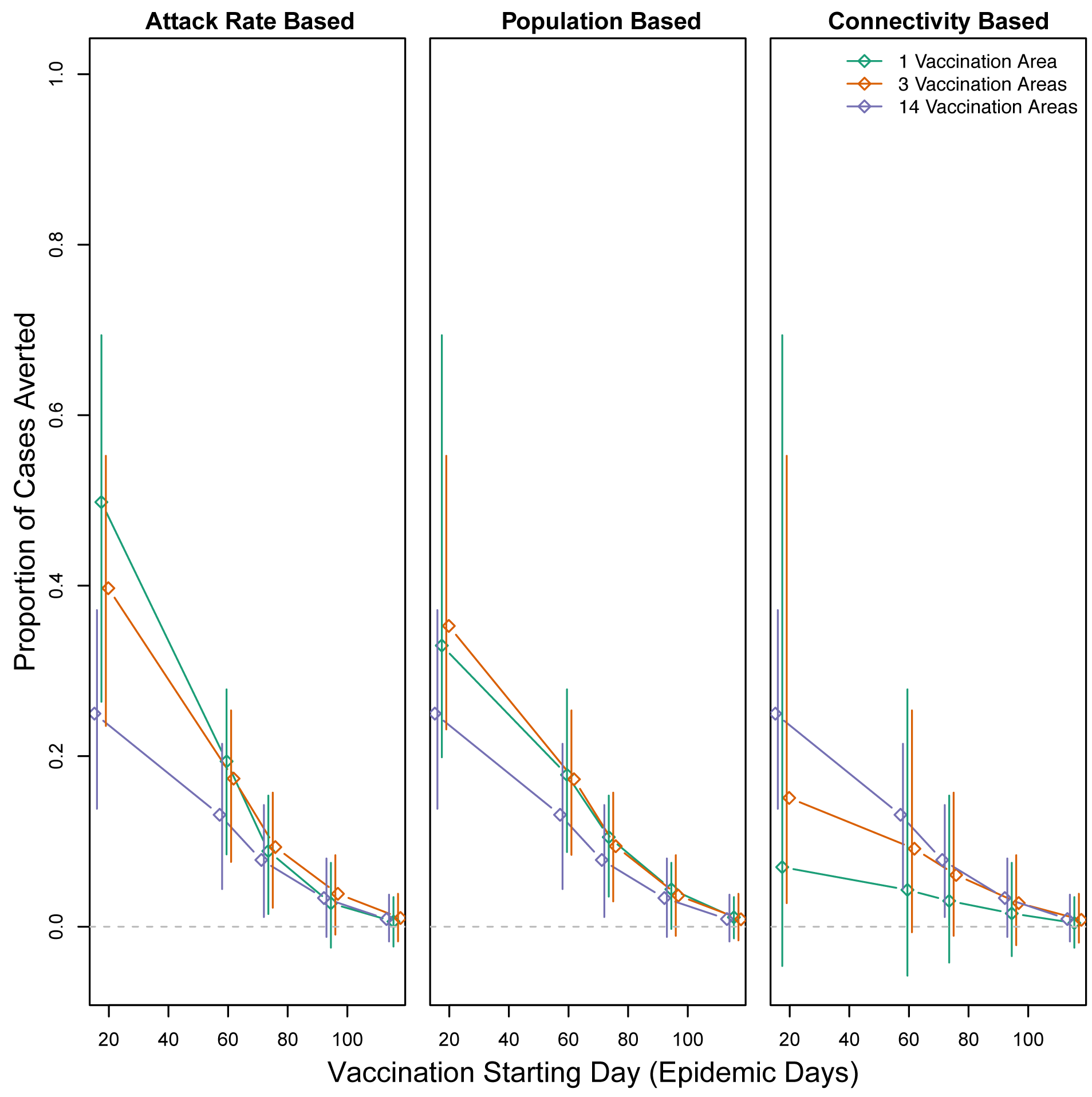

Supplement: Figure S7 — Vaccination simulation results with 7-day generation time, 75% vaccine efficacy, and 75,000 doses. (TIF) [file pntd.0001901.s007.tif]

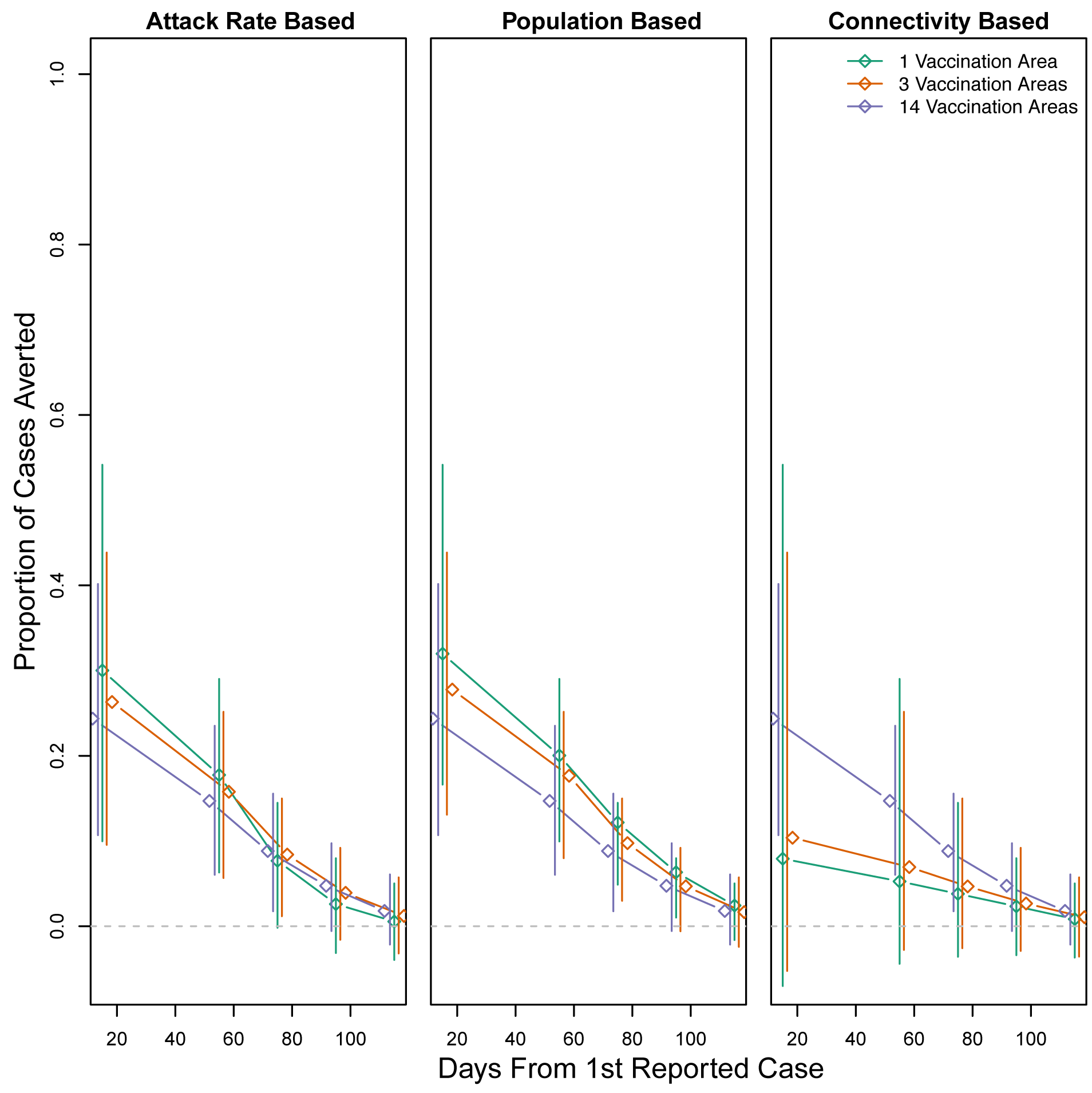

Supplement: Figure S8 — Vaccination simulation results with 10-day generation time, 75% vaccine efficacy, and 75,000 doses. (TIF) [file pntd.0001901.s008.tif]
