# Supplementary material for: Urban Cholera Transmission Hotspots and Their Implications for Reactive Vaccination: Evidence from Bissau City, Guinea Bissau
Source: PLoS Negl Trop Dis. 2012 Nov 8;6(11):e1901. doi: 10.1371/journal.pntd.0001901 (PMC3493445; doi:10.1371/journal.pntd.0001901)
Supplement: Table S1 — Vaccination simulation results with 50,000 doses and 75% vaccine efficacy. Proportion and number of cases averted in 5,000 simulations under different vaccination strategies (Median and 95% Predictive Interval). (DOCX) [file pntd.0001901.s009.docx]

|  | | **Vaccination Campaign Start Time** | | | | | | | |
| --- | --- | --- | --- | --- | --- | --- | --- | --- | --- |
| **Distribution** | **# Areas** | **Day 20** | | **Day 60** | | **Day 80** | | **Day 100** | |
| **Strategy** | **Vacc.** | **Cases** | **%** | **Cases** | **%** | **Cases** | **%** | **Cases** | **%** |
| **Attack Rate** | 1 | 3109 | 0.41 | 1828 | 0.23 | 739 | 0.09 | 256 | 0.03 |
|  |  | 1475,5198 | 0.21,0.69 | 757,2857 | 0.1,0.35 | -6,1523 | 0,0.18 | -266,791 | -0.03,0.09 |
|  | 2 | 2764 | 0.37 | 1678 | 0.22 | 732 | 0.09 | 276 | 0.03 |
|  |  | 1284,4491 | 0.18,0.6 | 618,2674 | 0.08,0.33 | -24,1475 | 0,0.18 | -243,829 | -0.03,0.1 |
|  | 3 | 2339 | 0.31 | 1441 | 0.19 | 708 | 0.09 | 317 | 0.04 |
|  |  | 1020,3770 | 0.14,0.49 | 507,2411 | 0.07,0.29 | -27,1451 | 0,0.17 | -199,853 | -0.02,0.1 |
| Population | 1 | 1328 | 0.18 | 935 | 0.12 | 556 | 0.07 | 259 | 0.03 |
|  |  | 158,2599 | 0.02,0.33 | 10,1928 | 0,0.24 | -208,1354 | -0.03,0.16 | -271,799 | -0.03,0.09 |
|  | 2 | 1476 | 0.2 | 1012 | 0.13 | 628 | 0.08 | 282 | 0.03 |
|  |  | 347,2715 | 0.05,0.34 | 69,2015 | 0.01,0.24 | -120,1370 | -0.02,0.16 | -237,819 | -0.03,0.1 |
|  | 3 | 2082 | 0.28 | 1386 | 0.18 | 727 | 0.09 | 294 | 0.04 |
|  |  | 871,3435 | 0.12,0.44 | 462,2334 | 0.06,0.28 | 4,1459 | 0,0.18 | -204,842 | -0.03,0.1 |
| Connectivity | 1 | 461 | 0.06 | 350 | 0.04 | 228 | 0.03 | 127 | 0.02 |
|  |  | -604,1499 | -0.09,0.19 | -565,1292 | -0.08,0.16 | -525,978 | -0.07,0.12 | -405,656 | -0.05,0.08 |
|  | 2 | 929 | 0.12 | 612 | 0.08 | 444 | 0.06 | 248 | 0.03 |
|  |  | -136,2059 | -0.02,0.26 | -311,1521 | -0.04,0.19 | -296,1149 | -0.04,0.14 | -262,776 | -0.03,0.09 |
|  | 3 | 1330 | 0.18 | 934 | 0.12 | 615 | 0.08 | 315 | 0.04 |
|  |  | 189,2486 | 0.03,0.31 | 14,1900 | 0,0.23 | -108,1362 | -0.01,0.16 | -203,845 | -0.03,0.1 |
| **Diffuse/** | 14 | 1538 | 0.21 | 1056 | 0.14 | 614 | 0.08 | 303 | 0.04 |
| **City-Wide** |  | 461,2734 | 0.07,0.34 | 177,1962 | 0.03,0.24 | -103,1378 | -0.01,0.16 | -207,844 | -0.03,0.1 |
