# Supplementary material for: Urban Cholera Transmission Hotspots and Their Implications for Reactive Vaccination: Evidence from Bissau City, Guinea Bissau
Source: PLoS Negl Trop Dis. 2012 Nov 8;6(11):e1901. doi: 10.1371/journal.pntd.0001901 (PMC3493445; doi:10.1371/journal.pntd.0001901)
Supplement: Table S2 — Vaccination simulation results with 100,000 doses and 75% vaccine efficacy. Proportion and number of cases averted in 5,000 simulations under different vaccination strategies (Median and 95% Predictive Interval). (DOCX) [file pntd.0001901.s010.docx]

|  | | **Vaccination Campaign Start Time** | | | | | | | |
| --- | --- | --- | --- | --- | --- | --- | --- | --- | --- |
| **Distribution** | **# Areas** | **Day 20** | | **Day 60** | | **Day 80** | | **Day 100** | |
| **Strategy** | **Vacc.** | **Cases** | **%** | **Cases** | **%** | **Cases** | **%** | **Cases** | **%** |
| **Attack Rate** | 1 | 4974 | 0.67 | 2732 | 0.35 | 1173 | 0.15 | 429 | 0.05 |
|  |  | 2947,6918 | 0.4,0.89 | 1630,3738 | 0.22,0.46 | 422,1906 | 0.06,0.23 | -66,975 | -0.01,0.11 |
|  | 2 | 4874 | 0.65 | 2680 | 0.34 | 1177 | 0.15 | 470 | 0.06 |
|  |  | 2752,6834 | 0.37,0.89 | 1558,3725 | 0.21,0.45 | 416,1924 | 0.05,0.23 | -42,986 | -0.01,0.11 |
|  | 3 | 4402 | 0.59 | 2496 | 0.32 | 1191 | 0.15 | 536 | 0.06 |
|  |  | 2662,6408 | 0.37,0.85 | 1492,3448 | 0.2,0.42 | 470,1942 | 0.06,0.23 | 26,1062 | 0,0.12 |
| Population | 1 | 2194 | 0.29 | 1536 | 0.2 | 924 | 0.12 | 431 | 0.05 |
|  |  | 957,3651 | 0.14,0.46 | 533,2550 | 0.07,0.31 | 147,1727 | 0.02,0.2 | -101,997 | -0.01,0.11 |
|  | 2 | 2418 | 0.32 | 1681 | 0.22 | 1017 | 0.13 | 483 | 0.06 |
|  |  | 1177,3888 | 0.17,0.5 | 725,2688 | 0.1,0.33 | 301,1832 | 0.04,0.22 | -34,1045 | 0,0.12 |
|  | 3 | 3888 | 0.52 | 2370 | 0.31 | 1221 | 0.15 | 510 | 0.06 |
|  |  | 2417,5599 | 0.33,0.72 | 1423,3334 | 0.2,0.4 | 493,1961 | 0.06,0.23 | 21,1075 | 0,0.12 |
| Connectivity | 1 | 1396 | 0.19 | 975 | 0.12 | 618 | 0.08 | 319 | 0.04 |
|  |  | 320,2503 | 0.05,0.32 | 39,1886 | 0.01,0.23 | -109,1368 | -0.01,0.16 | -197,866 | -0.02,0.1 |
|  | 2 | 1528 | 0.2 | 1007 | 0.13 | 697 | 0.09 | 400 | 0.05 |
|  |  | 393,2729 | 0.06,0.34 | 116,1968 | 0.02,0.24 | -30,1441 | 0,0.17 | -111,910 | -0.01,0.1 |
|  | 3 | 2144 | 0.29 | 1521 | 0.2 | 977 | 0.12 | 512 | 0.06 |
|  |  | 912,3528 | 0.13,0.45 | 575,2522 | 0.08,0.31 | 243,1754 | 0.03,0.21 | 27,1055 | 0,0.12 |
| **Diffuse/** | 14 | 2994 | 0.4 | 1943 | 0.25 | 1109 | 0.14 | 517 | 0.06 |
| **City-Wide** |  | 1911,4290 | 0.27,0.55 | 1031,2848 | 0.14,0.34 | 426,1847 | 0.06,0.22 | 51,1063 | 0.01,0.12 |

Table 2: **Simulation Results with 100,000 doses and 75% Vaccine Efficacy.** Proportion and number of cases averted in 5,000 simulations under different vaccination strategies (Median and 95% Predictive Interval). AR: Attack Rate Based Strategy, Pop: Population Based Targeting, Con: Connectivity Based, Dif: Diffuse (city-wide). signifies vaccination in 1 location with Attack Rate Based targeting.
