# Supplementary material for: Urban Cholera Transmission Hotspots and Their Implications for Reactive Vaccination: Evidence from Bissau City, Guinea Bissau
Source: PLoS Negl Trop Dis. 2012 Nov 8;6(11):e1901. doi: 10.1371/journal.pntd.0001901 (PMC3493445; doi:10.1371/journal.pntd.0001901)
Supplement: Table S3 — Vaccination simulation results from 3-day generation time model, 75,000 doses. Proportion and number of cases averted in 5,000 simulations under different vaccination strategies (Median and 95% Predictive Interval). (DOCX) [file pntd.0001901.s011.docx]

|  | | **Vaccination Campaign Start Time** | | | | | | | |
| --- | --- | --- | --- | --- | --- | --- | --- | --- | --- |
| **Distribution** | **# Areas** | **Day 20** | | **Day 60** | | **Day 80** | | **Day 100** | |
| **Strategy** | **Vacc.** | **Cases** | **%** | **Cases** | **%** | **Cases** | **%** | **Cases** | **%** |
| **Attack Rate** | 1 | 5947 | 0.79 | 4503 | 0.48 | 3281 | 0.3 | 2458 | 0.22 |
|  |  | 1764,9507 | 0.39,0.94 | 2215,7387 | 0.28,0.63 | 1474,5582 | 0.15,0.43 | 804,4452 | 0.08,0.35 |
|  | 2 | 5770 | 0.77 | 4461 | 0.47 | 3280 | 0.3 | 2482 | 0.23 |
|  |  | 1781,9429 | 0.4,0.94 | 2234,7478 | 0.28,0.63 | 1553,5601 | 0.16,0.43 | 827,4530 | 0.09,0.35 |
|  | 3 | 5068 | 0.66 | 3812 | 0.4 | 2779 | 0.25 | 2098 | 0.19 |
|  |  | 1069,8924 | 0.25,0.92 | 1502,6594 | 0.19,0.57 | 1060,5061 | 0.11,0.39 | 424,4059 | 0.04,0.32 |
| Population | 1 | 2280 | 0.29 | 1932 | 0.2 | 1608 | 0.15 | 1206 | 0.11 |
|  |  | -1567,6805 | -0.39,0.85 | -508,4516 | -0.07,0.41 | -239,3834 | -0.02,0.3 | -521,3180 | -0.05,0.26 |
|  | 2 | 1820 | 0.23 | 1477 | 0.15 | 1250 | 0.11 | 950 | 0.09 |
|  |  | -2006,6210 | -0.46,0.8 | -830,4101 | -0.1,0.37 | -609,3326 | -0.06,0.26 | -839,2907 | -0.09,0.24 |
|  | 3 | 4363 | 0.56 | 3253 | 0.34 | 2465 | 0.22 | 1817 | 0.17 |
|  |  | 515,8363 | 0.12,0.92 | 1002,6096 | 0.13,0.53 | 731,4690 | 0.08,0.36 | 163,3781 | 0.02,0.3 |
| Connectivity | 1 | 572 | 0.07 | 490 | 0.05 | 425 | 0.04 | 324 | 0.03 |
|  |  | -3590,4652 | -0.83,0.59 | -1953,3032 | -0.23,0.27 | -1502,2428 | -0.15,0.2 | -1559,2279 | -0.16,0.19 |
|  | 2 | 1100 | 0.14 | 976 | 0.1 | 794 | 0.07 | 602 | 0.05 |
|  |  | -2765,5301 | -0.63,0.71 | -1500,3517 | -0.19,0.32 | -1081,2760 | -0.11,0.23 | 1167,2557 | -0.12,0.21 |
|  | 3 | 1314 | 0.17 | 1115 | 0.12 | 906 | 0.08 | 728 | 0.07 |
|  |  | -3091,5534 | -0.96,0.73 | -1281,3642 | -0.16,0.33 | -951,2946 | -0.09,0.24 | 1127,2650 | -0.11,0.21 |
| **Diffuse/** | 14 | 4686 | 0.61 | 3449 | 0.36 | 2662 | 0.24 | 2090 | 0.19 |
| **City-Wide** |  | 787,8643 | 0.16,0.93 | 1221,6131 | 0.16,0.54 | 917,4703 | 0.09,0.37 | 446,4131 | 0.05,0.32 |

Table 3: Vaccine Simulation Results from 3-day generation time model, 75,00 doses
