# Supplementary material for: Urban Cholera Transmission Hotspots and Their Implications for Reactive Vaccination: Evidence from Bissau City, Guinea Bissau
Source: PLoS Negl Trop Dis. 2012 Nov 8;6(11):e1901. doi: 10.1371/journal.pntd.0001901 (PMC3493445; doi:10.1371/journal.pntd.0001901)
Supplement: Table S4 — Vaccination simulation results from 7-day generation time model, 75,000 doses. Proportion and number of cases averted in 5,000 simulations under different vaccination strategies (Median and 95% Predictive Interval). (DOCX) [file pntd.0001901.s012.docx]

|  | | **Vaccination Campaign Start Time** | | | | | | | |
| --- | --- | --- | --- | --- | --- | --- | --- | --- | --- |
| **Distribution** | **# Areas** | **Day 20** | | **Day 60** | | **Day 80** | | **Day 100** | |
| **Strategy** | **Vacc.** | **Cases** | **%** | **Cases** | **%** | **Cases** | **%** | **Cases** | **%** |
| **Attack Rate** | 1 | 3788 | 0.51 | 1518 | 0.25 | 709 | 0.14 | 235 | 0.07 |
|  |  | 1923,5360 | 0.26,0.72 | 624,2305 | 0.11,0.36 | 50,1343 | 0.01,0.24 | -244,717 | -0.08,0.19 |
|  | 2 | 3324 | 0.45 | 1418 | 0.24 | 709 | 0.13 | 255 | 0.08 |
|  |  | 1786,4808 | 0.25,0.64 | 555,2190 | 0.1,0.34 | 38,1330 | 0.01,0.24 | -221,726 | -0.07,0.2 |
|  | 3 | 3029 | 0.41 | 1364 | 0.23 | 752 | 0.14 | 323 | 0.09 |
|  |  | 1715,4276 | 0.24,0.57 | 521,2130 | 0.09,0.33 | 93,1374 | 0.02,0.25 | -146,797 | -0.05,0.21 |
| Population | 1 | 2518 | 0.34 | 1394 | 0.23 | 856 | 0.16 | 366 | 0.11 |
|  |  | 1400,4027 | 0.2,0.52 | 615,2326 | 0.11,0.36 | 223,1599 | 0.05,0.28 | -83,903 | -0.03,0.23 |
|  | 2 | 1985 | 0.27 | 1146 | 0.19 | 714 | 0.14 | 306 | 0.09 |
|  |  | 995,3387 | 0.14,0.44 | 391,2031 | 0.07,0.31 | 84,1430 | 0.02,0.25 | -132,828 | -0.04,0.22 |
|  | 3 | 2680 | 0.36 | 1359 | 0.23 | 776 | 0.15 | 307 | 0.09 |
|  |  | 1652,3933 | 0.24,0.52 | 611,2181 | 0.11,0.34 | 155,1465 | 0.03,0.25 | -148,823 | -0.05,0.21 |
| Connectivity | 1 | 907 | 0.12 | 495 | 0.08 | 365 | 0.07 | 188 | 0.05 |
|  |  | -107,1913 | -0.02,0.25 | -299,1281 | -0.05,0.2 | -294,1023 | -0.06,0.18 | -289,675 | -0.09,0.18 |
|  | 2 | 2576 | 0.35 | 1412 | 0.23 | 908 | 0.17 | 399 | 0.12 |
|  |  | 1519,4001 | 0.22,0.52 | 670,2336 | 0.12,0.36 | 278,1640 | 0.06,0.28 | -52,912 | -0.02,0.24 |
|  | 3 | 2439 | 0.33 | 1389 | 0.23 | 883 | 0.17 | 388 | 0.11 |
|  |  | 1432,3832 | 0.21,0.5 | 642,2284 | 0.12,0.35 | 270,1585 | 0.06,0.27 | -58,899 | -0.02,0.23 |
| **Diffuse/** | 14 | 1896 | 0.26 | 1025 | 0.17 | 635 | 0.12 | 285 | 0.08 |
| **City-Wide** |  | 965,2911 | 0.14,0.38 | 271,1805 | 0.05,0.28 | 26,1282 | 0.01,0.22 | -160,770 | -0.05,0.21 |

Table 4: Vaccine Simulation Results from 7-day generation time model, 75,000 doses
