# Supplementary material for: Urban Cholera Transmission Hotspots and Their Implications for Reactive Vaccination: Evidence from Bissau City, Guinea Bissau
Source: PLoS Negl Trop Dis. 2012 Nov 8;6(11):e1901. doi: 10.1371/journal.pntd.0001901 (PMC3493445; doi:10.1371/journal.pntd.0001901)
Supplement: Table S5 — Vaccination simulation results from 10-day generation time model. Proportion and number of cases averted in 5,000 simulations under different vaccination strategies (Median and 95% Predictive Interval). (DOCX) [file pntd.0001901.s013.docx]

|  | | **Vaccination Campaign Start Time** | | | | | | | |
| --- | --- | --- | --- | --- | --- | --- | --- | --- | --- |
| **Distribution** | **# Areas** | **Day 20** | | **Day 60** | | **Day 80** | | **Day 100** | |
| **Strategy** | **Vacc.** | **Cases** | **%** | **Cases** | **%** | **Cases** | **%** | **Cases** | **%** |
| **Attack Rate** | 1 | 2346 | 0.3 | 1497 | 0.18 | 674 | 0.08 | 233 | 0.03 |
|  |  | 740,4201 | 0.1,0.54 | 507,2489 | 0.06,0.29 | -16,1314 | 0,0.14 | -274,733 | -0.03,0.08 |
|  | 2 | 2668 | 0.34 | 1697 | 0.2 | 878 | 0.1 | 397 | 0.04 |
|  |  | 1136,4257 | 0.16,0.54 | 788,2599 | 0.1,0.3 | 204,1544 | 0.02,0.17 | -105,883 | -0.01,0.1 |
|  | 3 | 2068 | 0.26 | 1334 | 0.16 | 736 | 0.08 | 352 | 0.04 |
|  |  | 676,3448 | 0.1,0.44 | 449,2166 | 0.06,0.25 | 101,1369 | 0.01,0.15 | -137,850 | -0.02,0.09 |
| Population | 1 | 2502 | 0.32 | 1687 | 0.2 | 1067 | 0.12 | 570 | 0.06 |
|  |  | 1217,3989 | 0.17,0.5 | 793,2587 | 0.1,0.29 | 408,1775 | 0.05,0.19 | 90,1082 | 0.01,0.12 |
|  | 2 | 1937 | 0.25 | 1356 | 0.16 | 869 | 0.1 | 472 | 0.05 |
|  |  | 713,3323 | 0.1,0.41 | 530,2209 | 0.07,0.25 | 227,1543 | 0.03,0.17 | -12,1000 | 0,0.11 |
|  | 3 | 2187 | 0.28 | 1487 | 0.18 | 852 | 0.1 | 421 | 0.05 |
|  |  | 955,3546 | 0.13,0.45 | 645,2326 | 0.08,0.27 | 249,1491 | 0.03,0.16 | -52,912 | -0.01,0.1 |
| Connectivity | 1 | 620 | 0.08 | 443 | 0.05 | 332 | 0.04 | 208 | 0.02 |
|  |  | -487,1766 | -0.07,0.22 | -349,1255 | -0.04,0.14 | -302,984 | -0.04,0.11 | -296,729 | -0.03,0.08 |
|  | 2 | 712 | 0.09 | 501 | 0.06 | 372 | 0.04 | 233 | 0.03 |
|  |  | -399,1855 | -0.06,0.23 | -323,1329 | -0.04,0.15 | -289,1002 | -0.04,0.11 | -284,738 | -0.03,0.08 |
|  | 3 | 811 | 0.1 | 582 | 0.07 | 409 | 0.05 | 239 | 0.03 |
|  |  | -378,1984 | -0.05,0.24 | -215,1397 | -0.03,0.16 | -216,1037 | -0.03,0.11 | -252,756 | -0.03,0.08 |
| **Diffuse/** | 14 | 1911 | 0.24 | 1241 | 0.15 | 770 | 0.09 | 425 | 0.05 |
| **City-Wide** |  | 783,3206 | 0.11,0.4 | 483,2062 | 0.06,0.24 | 148,1432 | 0.02,0.16 | -49,916 | -0.01,0.1 |

Table 5: Vaccine Simulation Results from 10-day generation time model
