# Supplementary material for: Urban Cholera Transmission Hotspots and Their Implications for Reactive Vaccination: Evidence from Bissau City, Guinea Bissau
Source: PLoS Negl Trop Dis. 2012 Nov 8;6(11):e1901. doi: 10.1371/journal.pntd.0001901 (PMC3493445; doi:10.1371/journal.pntd.0001901)
Supplement: Table S6 — Vaccination simulation results with 75,000 doses and 65% vaccine efficacy. Proportion and number of cases averted in 5,000 simulations under different vaccination strategies (Median and 95% Predictive Interval). (DOCX) [file pntd.0001901.s014.docx]

|  | | **Vaccination Campaign Start Time** | | | | | | | |
| --- | --- | --- | --- | --- | --- | --- | --- | --- | --- |
| **Distribution** | **# Areas** | **Day 20** | | **Day 60** | | **Day 80** | | **Day 100** | |
| **Strategy** | **Vacc.** | **Cases** | **%** | **Cases** | **%** | **Cases** | **%** | **Cases** | **%** |
| **Attack Rate** | 1 | 3826 | 0.51 | 2164 | 0.28 | 883 | 0.11 | 316 | 0.04 |
|  |  | 1981,6138 | 0.27,0.82 | 1044,3216 | 0.14,0.39 | 122,1629 | 0.02,0.19 | -226,872 | -0.03,0.1 |
|  | 2 | 3508 | 0.47 | 2037 | 0.26 | 898 | 0.11 | 344 | 0.04 |
|  |  | 1831,5545 | 0.25,0.73 | 947,3056 | 0.13,0.37 | 145,1648 | 0.02,0.2 | -161,881 | -0.02,0.1 |
|  | 3 | 2997 | 0.4 | 1802 | 0.23 | 869 | 0.11 | 389 | 0.05 |
|  |  | 1581,4705 | 0.22,0.62 | 780,2808 | 0.11,0.34 | 134,1615 | 0.02,0.19 | -126,926 | -0.02,0.11 |
| Population | 1 | 1635 | 0.22 | 1128 | 0.15 | 690 | 0.09 | 317 | 0.04 |
|  |  | 432,2978 | 0.06,0.38 | 157,2159 | 0.02,0.26 | -83,1499 | -0.01,0.18 | -197,880 | -0.02,0.1 |
|  | 2 | 1802 | 0.24 | 1263 | 0.16 | 762 | 0.1 | 349 | 0.04 |
|  |  | 648,3086 | 0.09,0.39 | 310,2219 | 0.04,0.27 | 50,1547 | 0.01,0.18 | -168,906 | -0.02,0.1 |
|  | 3 | 2671 | 0.35 | 1710 | 0.22 | 890 | 0.11 | 375 | 0.05 |
|  |  | 1393,4135 | 0.2,0.53 | 776,2698 | 0.11,0.32 | 136,1639 | 0.02,0.19 | -129,910 | -0.02,0.1 |
| Connectivity | 1 | 616 | 0.08 | 418 | 0.05 | 302 | 0.04 | 162 | 0.02 |
|  |  | -441,1703 | -0.06,0.21 | -483,1351 | -0.07,0.16 | -452,1033 | -0.06,0.12 | -378,698 | -0.05,0.08 |
|  | 2 | 1152 | 0.15 | 768 | 0.1 | 522 | 0.07 | 298 | 0.04 |
|  |  | 45,2284 | 0.01,0.29 | -124,1702 | -0.02,0.21 | -199,1268 | -0.03,0.15 | -214,827 | -0.03,0.1 |
|  | 3 | 1604 | 0.21 | 1128 | 0.14 | 759 | 0.1 | 383 | 0.05 |
|  |  | 449,2824 | 0.07,0.36 | 201,2132 | 0.03,0.26 | 24,1510 | 0,0.18 | -120,909 | -0.02,0.1 |
| **Diffuse/** | 14 | 1987 | 0.26 | 1351 | 0.17 | 778 | 0.1 | 375 | 0.05 |
| **City-Wide** |  | 961,3168 | 0.14,0.4 | 494,2245 | 0.07,0.27 | 70,1531 | 0.01,0.18 | -141,920 | -0.02,0.11 |

Table 6: **Simulation Results with 75,000 doses and 65% Vaccine Efficacy.**
