# Supplementary material for: Urban Cholera Transmission Hotspots and Their Implications for Reactive Vaccination: Evidence from Bissau City, Guinea Bissau
Source: PLoS Negl Trop Dis. 2012 Nov 8;6(11):e1901. doi: 10.1371/journal.pntd.0001901 (PMC3493445; doi:10.1371/journal.pntd.0001901)
Supplement: Table S7 — Vaccination simulation results with 75,000 doses and 85% vaccine efficacy. Proportion and number of cases averted in 5,000 simulations under different vaccination strategies (Median and 95% Predictive Interval). (DOCX) [file pntd.0001901.s015.docx]

|  | | **Vaccination Campaign Start Time** | | | | | | | |
| --- | --- | --- | --- | --- | --- | --- | --- | --- | --- |
| **Distribution** | **# Areas** | **Day 20** | | **Day 60** | | **Day 80** | | **Day 100** | |
| **Strategy** | **Vacc.** | **Cases** | **%** | **Cases** | **%** | **Cases** | **%** | **Cases** | **%** |
| **Attack Rate** | 1 | 4564 | 0.61 | 2506 | 0.32 | 1046 | 0.13 | 372 | 0.05 |
|  |  | 2476,6625 | 0.33,0.87 | 1330,3521 | 0.18,0.43 | 275,1789 | 0.04,0.22 | -163,905 | -0.02,0.11 |
|  | 2 | 4403 | 0.59 | 2434 | 0.31 | 1068 | 0.14 | 415 | 0.05 |
|  |  | 2483,6490 | 0.34,0.85 | 1317,3434 | 0.18,0.42 | 323,1806 | 0.04,0.22 | -87,950 | -0.01,0.11 |
|  | 3 | 3845 | 0.51 | 2223 | 0.29 | 1079 | 0.14 | 474 | 0.06 |
|  |  | 2254,5820 | 0.31,0.77 | 1212,3188 | 0.17,0.39 | 358,1816 | 0.05,0.22 | -34,979 | 0,0.11 |
| Population | 1 | 1970 | 0.26 | 1382 | 0.18 | 826 | 0.11 | 379 | 0.05 |
|  |  | 739,3367 | 0.1,0.43 | 392,2411 | 0.05,0.29 | 83,1652 | 0.01,0.19 | -157,939 | -0.02,0.11 |
|  | 2 | 2184 | 0.29 | 1496 | 0.19 | 927 | 0.12 | 426 | 0.05 |
|  |  | 987,3534 | 0.14,0.45 | 558,2495 | 0.08,0.3 | 186,1736 | 0.03,0.2 | -92,995 | -0.01,0.11 |
|  | 3 | 3364 | 0.45 | 2126 | 0.27 | 1088 | 0.14 | 464 | 0.06 |
|  |  | 1999,5044 | 0.28,0.65 | 1194,3131 | 0.16,0.38 | 365,1835 | 0.05,0.22 | -23,996 | 0,0.11 |
| Connectivity | 1 | 707 | 0.09 | 520 | 0.07 | 348 | 0.04 | 191 | 0.02 |
|  |  | -349,1780 | -0.05,0.22 | -395,1448 | -0.06,0.18 | -412,1118 | -0.06,0.13 | -323,730 | -0.04,0.09 |
|  | 2 | 1360 | 0.18 | 910 | 0.12 | 632 | 0.08 | 357 | 0.04 |
|  |  | 277,2551 | 0.04,0.32 | -26,1881 | 0,0.23 | -117,1380 | -0.02,0.16 | -140,902 | -0.02,0.1 |
|  | 3 | 1946 | 0.26 | 1360 | 0.18 | 916 | 0.12 | 449 | 0.05 |
|  |  | 748,3244 | 0.11,0.41 | 409,2390 | 0.06,0.29 | 180,1662 | 0.02,0.2 | -39,965 | 0,0.11 |
| **Diffuse/** | 14 | 2570 | 0.34 | 1684 | 0.22 | 978 | 0.12 | 464 | 0.06 |
| **City-Wide** |  | 1430,3849 | 0.2,0.49 | 790,2602 | 0.11,0.31 | 305,1721 | 0.04,0.2 | -31,991 | 0,0.11 |

Table 7: **Simulation Results with 75,000 doses and 85% Vaccine Efficacy.** Proportion and number of cases averted in 5,000 simulations under different vaccination strategies (Median and 95% Predictive Interval). AR: Attack Rate Based Strategy, Pop: Population Based Targeting, Con: Connectivity Based, Dif: Diffuse (city-wide). signifies vaccination in 1 location with Attack Rate Based targeting.
