# Supplementary material for: Urban Cholera Transmission Hotspots and Their Implications for Reactive Vaccination: Evidence from Bissau City, Guinea Bissau
Source: PLoS Negl Trop Dis. 2012 Nov 8;6(11):e1901. doi: 10.1371/journal.pntd.0001901 (PMC3493445; doi:10.1371/journal.pntd.0001901)
Supplement: Text S1 — Details on final model, model selection, and simulations. (PDF) [file pntd.0001901.s016.pdf]

Urban Cholera Transmission Hotspots and their Implications for Reactive Vaccination: Evidence from Bissau City, Guinea Bissau
Supplemental Text 1 - Model Selection/Diagnostics

Andrew Azman, Francisco J. Luquero, Amabelia Rodrigues, Rebecca F. Grais, Cunhate Na Banga, Pedro Pablo Palma, Bryan T. Grenfell, Justin Lessler

1 Model Selection

We generated a series of models with different assumptions about the form of the internal and external transmission coefficients. The sections below describes the form of each model. In all cases we used non-informative prior distributions. Table 1 shows the Deviance Information Criteria (DIC) for each model described below.

All models were fit in JAGS 3.0 [1]. We ran three chains for each model and assessed convergence by monitoring $\hat{R}$ with a threshold of 1.01 in addition to visual confirmation of the trace plots of all three chains. We ran models until convergence (ranging from 100,000 - 350,000 iterations), saved every 20-50th iteration depending on the number of iterations.

The following model forms the basis of each model shown below:

$$I_{i,t+1} \sim Poisson\left(\frac{S_{i,t}(\phi)}{N_i}(\beta_i I_{i,t} + \sum_{j\neq i} I_{j,t}\alpha_{j,i})\right)$$

where:
$I_{i,t}$: Number of observed incident cases in area i at time t
$S_{i,t}$: Number of susceptible individuals in area i at time t
N_i: Population size in area i
β_i: Internal transmission coefficient for area i
$\alpha_{j,i}$: External transmission coefficient for a new case in area i being infected as a result of a case in area j
ϕ: Fraction of infectious individuals who are symptomatic and present to cholera treatment facilities within the city

After each step in the model we update the number of susceptibles with the following rule:

$$S_{i,t+1}(\phi) = S_{i,t}(\phi) - \frac{I_{i,t}}{\phi}$$

1.0.1 Model 1.1

In this model we only allowed for two unique transmission parameters; one for internal transmission (β) and one for external transmission (i.e. between areas, α). We assigned non-informative normal priors centered at zero ($N(0, \frac{1}{0.001})$) for natural logarithm of both of these parameters.

1.0.2 Model 1.2

To relax Model 1.1, we allowed for each location to have an independent internal transmission coefficient (β_i) and only allowed for one external transmission coefficient shared by all locations (α). The natural logarithm of all transmission coefficients here were assigned independent $N(0, \frac{1}{0.001})$ priors.

1.0.3 Model 1.3

In this model we allow for each location have its own internal transmission coefficient (β_i), and have symmetric external transmission coefficients (i.e. $\alpha_{i,j} = \alpha_{j,i}$). We put separate diffuse normal hyper-priors on the mean and variance of external and internal coefficients.

1.0.4 Model 1.4 (Model Used in Paper)

Next we relaxed the symmetry assumption and allowed each location to have its own unique internal and external transmission coefficients. The coefficients are linked through independent normal hyperpriors; one for internal and another for external transmission coefficients. This is the model presented in the paper:

$$
\begin{aligned}
I_{i,t+1} &\sim Poisson\left(\frac{S_{i,t}(\phi)}{N_i}(\beta_i I_{i,t} + \sum_{j\neq i} I_{j,t}\alpha_{j,i})\right) \\
\log(\alpha_{j,i}) &\sim N(\alpha_0, \tau_{0,\alpha}) \\
\log(\beta_i) &\sim N(\beta_0, \tau_{0,\beta}) \\
\alpha_0 &\sim N(0, \frac{1}{0.001}) \\
\beta_0 &\sim N(0, \frac{1}{0.001}) \\
\sigma_{0,\alpha} &\sim U(0, 1.5) \\
\tau_{0,\alpha} &= \frac{1}{\sigma^2_{0,\alpha}} \\
\sigma_{0,\beta} &\sim U(0, 1.5) \\
\tau_{0,\beta} &= \frac{1}{\sigma^2_{0,\beta}} \\
logit(\phi) &\sim N(0, \frac{1}{0.01})
\end{aligned}
$$

1.0.5 Models with Seasonality

Since cholera transmission tends to vary seasonally [2, 3, 4], we used data from all epidemics in SAB since 1997 (with data from the epidemic in 2001-2002 missing) to estimate the seasonal force of infection. We used a generalized additive model with only day of year as an independent variable. This model was fit in R using the "gam" package [5] with the degree of smoothness determined by generalized cross validation. We centered the seasonal function at 0 and rescaled it lie between -1 and +1 as shown in Figure 1. Seasonal forcing was incorporated into the model as follows:

$$
I_{i,t+1} \sim Poisson\left((1 + \beta_{seas} f_{seas}(t))\frac{S_{i,t}(\phi)}{N_i}(\beta_i I_{i,t} + \sum_{j\neq i} I_{j,t}\alpha_{j,i})\right)
$$

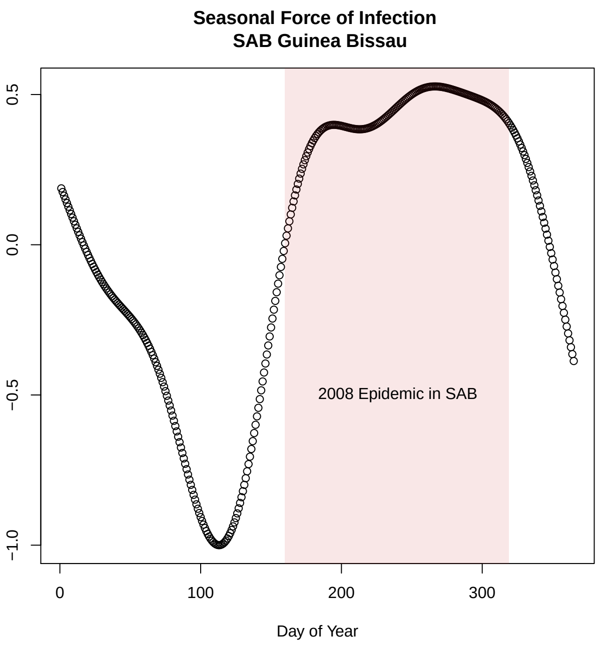

Figure 1: Scaled and centered seasonal forcing function fit using a generalized additive model

Table 1: Comparison of models with and without seasonality (s)

	Model 1.1	Model 1.2	Model 1.3	**Model 1.4**	Model 1.1s	Model 1.2s	Model 1.3s	Model 1.4s
DIC	3514.10	3118.90	2997.20	2957.30	3514.50	3133.10	2998.00	2956.90
Marginal Mean Posterior Internal Transmission Parameters								
$\log(\beta_1)$	-0.04	-0.29	-1.20	-2.92	-0.04	-0.28	-1.18	-3.03
$\log(\beta_2)$	-0.04	-0.33	-2.91	-4.38	-0.04	-0.32	-2.92	-4.45
$\log(\beta_3)$	-0.04	-0.02	-1.02	-1.21	-0.04	-0.02	-1.02	-1.28
$\log(\beta_4)$	-0.04	-0.42	-0.27	-0.27	-0.04	-0.40	-0.28	-0.36
$\log(\beta_5)$	-0.04	-27.50	-3.27	-4.61	-0.04	-3.10	-3.27	-4.71
$\log(\beta_6)$	-0.04	-0.63	-2.08	-3.88	-0.04	-0.60	-2.06	-4.02
$\log(\beta_7)$	-0.04	-27.95	-3.11	-4.51	-0.04	-3.11	-3.10	-4.61
$\log(\beta_8)$	-0.04	0.34	0.28	0.16	-0.04	0.34	0.27	0.04
$\log(\beta_9)$	-0.04	-0.47	-0.59	-0.80	-0.04	-0.46	-0.59	-0.92
$\log(\beta_{10})$	-0.04	-1.16	-1.27	-1.32	-0.04	-1.12	-1.27	-1.43
$\log(\beta_{11})$	-0.04	-0.81	-0.77	-0.81	-0.04	-0.78	-0.78	-0.93
$\log(\beta_{12})$	-0.04	-0.50	-1.28	-2.66	-0.04	-0.48	-1.28	-2.73
$\log(\beta_{13})$	-0.04	-0.06	-1.09	-0.90	-0.04	-0.06	-1.08	-1.02
$\log(\beta_{14})$	-0.04	-0.09	-0.41	-0.94	-0.04	-0.09	-0.42	-1.05

Note: The difference in DIC between Models 1.4 and 1.4s is not practically significant therefore we chose to use the simpler of the two models, 1.4.

1.1 Epidemic Simulations and Prediction Intervals

Each epidemic simulation was performed by drawing a new set of parameters from the joint posterior distribution then stepping through time and drawing new cases from a Poisson distribution as specified in 1.0.4. Ninety five percent prediction intervals were calculated by taking the 2.5 and 97.5 percentiles at each time step.

1.2 Model Fit

Figure 2 illustrates the posterior mean transmission coefficients and their standard deviation from the final model from 5,000 simulations.

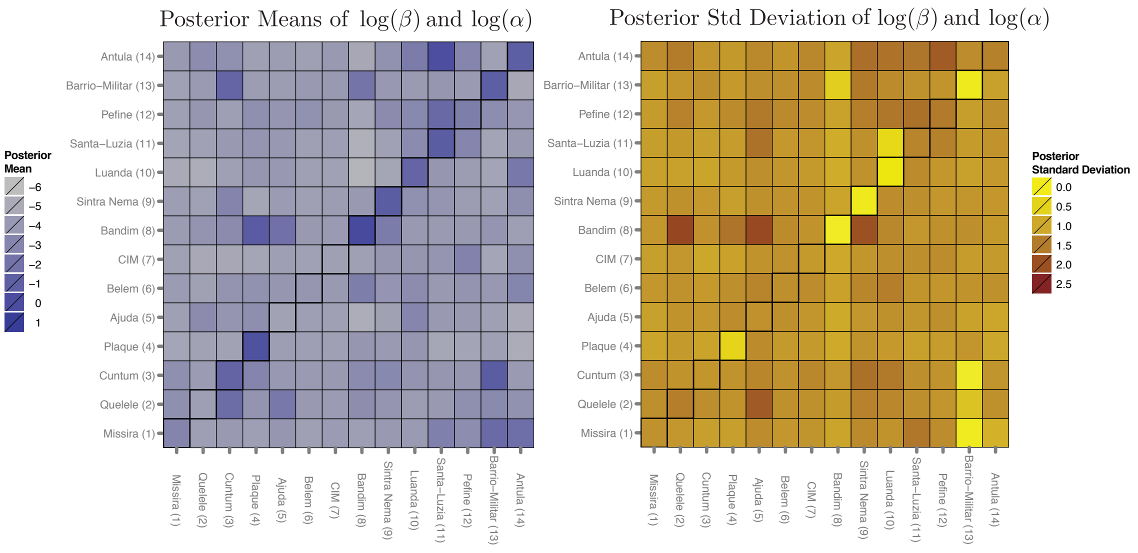

Figure 2: Posterior means for $log(\beta_i)$ (diagnonal) and $log(\alpha_{j,i})$) (off-diagonal) for each location from Model 1.4. For example $\alpha_{2,8}$, read from column 2 row 8, can be interpreted as the tranmission coefficient for cases arising in area 8 from prevalent cases in area 2.

We assessed model fit by looking at the mean squared error and coverage of 95% prediction intervals from n-step ahead predictions. We ran full simulations starting from the initial conditions of the 2008 epidemic and also looked at the fit from starting later points in time (Figure 6). Figures 3 and 4 show one and three step-ahead predictions along with Figure 5, which illustrates simulations of the full epidemic.

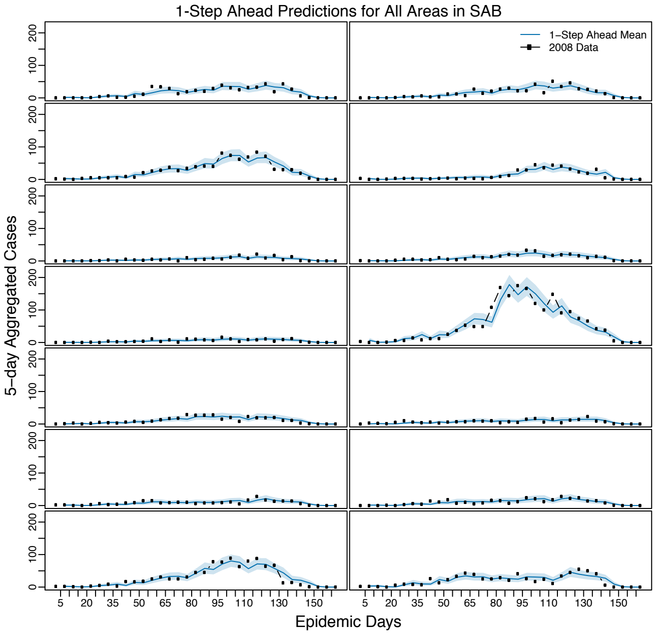

Figure 3: 1-step ahead predictions for all areas and 95% prediction intervals. Data from the 2008 epidemic shown in black

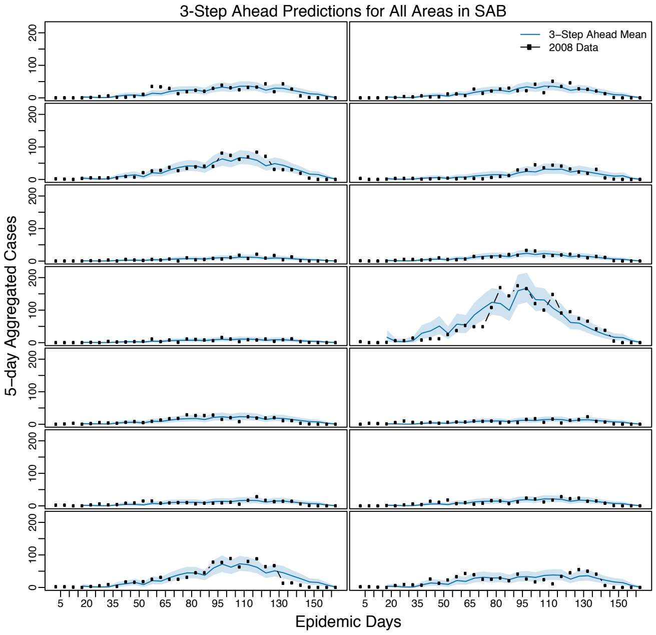

Figure 4: 3 step ahead predictions for all areas

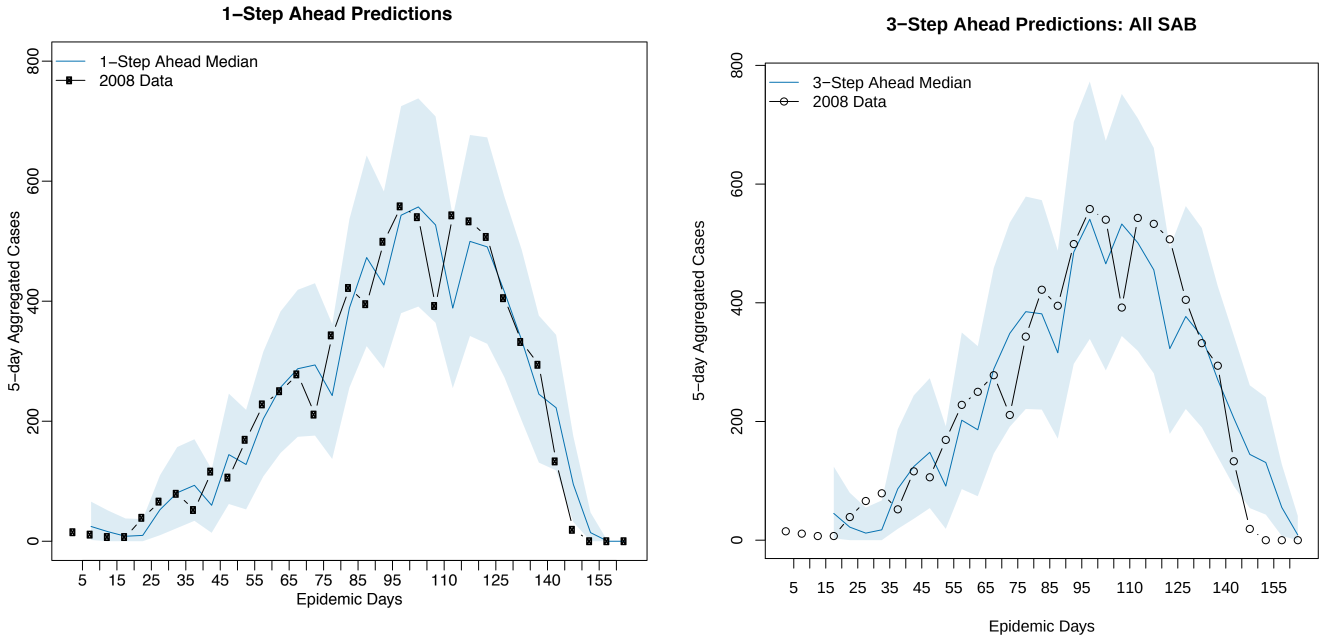

Figure 5: SAB-wide 1 (left) and 3 (right) Step Ahead Predictions. MSEs are 3114.8 and 5376.9, respectively. 95% Credible Interval coverage equals 81.3% and 76.7%, respectively. 95% CI coverage for 10 step ahead predictions shown in paper is 82.3% with an MSE of 10781.3.

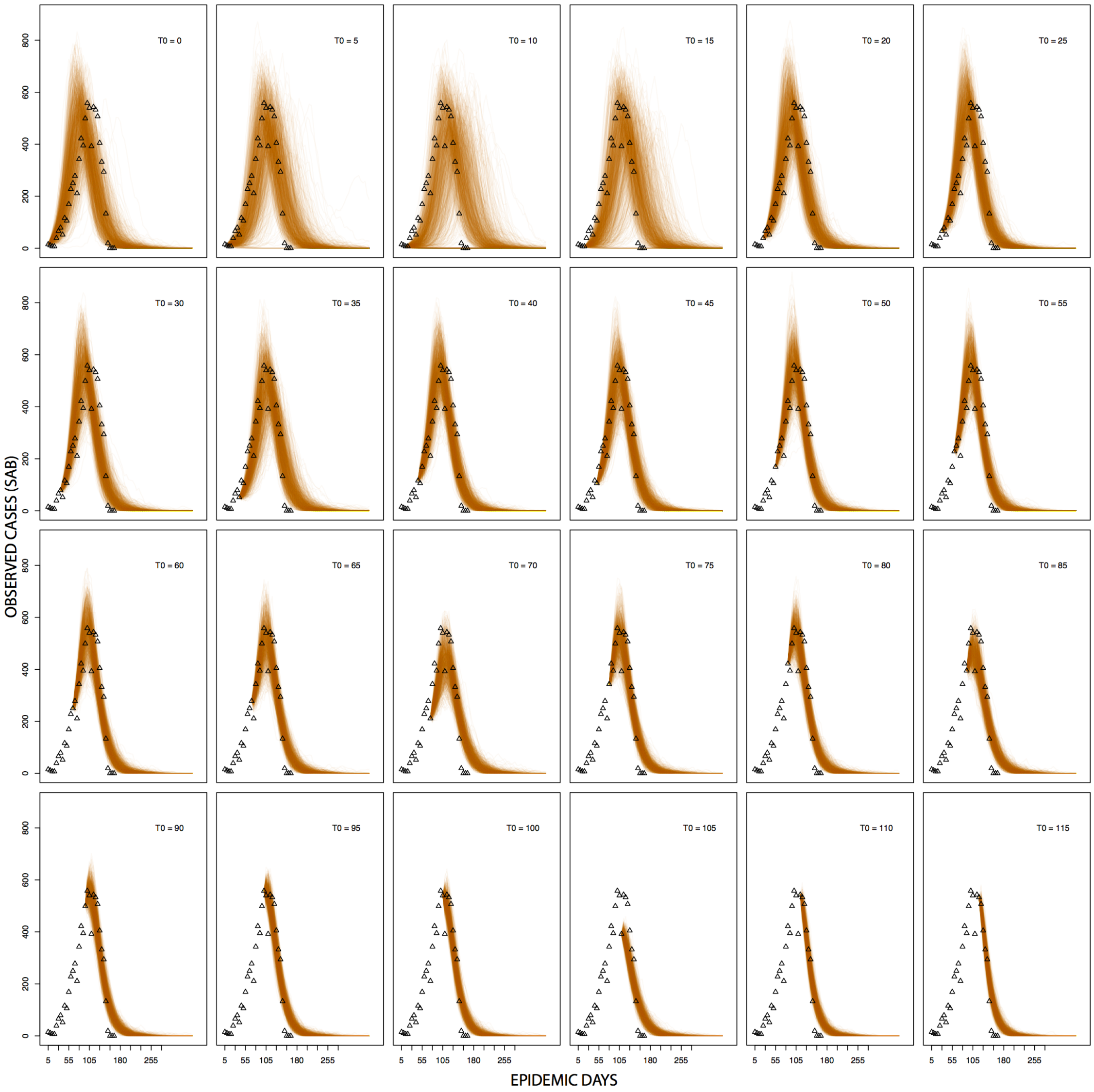

Figure 6: Full prediction for all areas starting from observed cases at different starting times (T0 represents first epidemic day of simulation). 10,000 simulations shown in orange with observed data shown as triangles.

1.3 Transmission Dynamics

We determined the sanitary area responsible for each new case at each time step, with the new cases in area i caused by area j at time $t+1$ defined by:

$$\begin{cases} \frac{S_{i,t}(\phi)}{N_i}\beta_i I_{i,t} & \text{if } i = j \\ \frac{S_{i,t}(\phi)}{N_i}\alpha_{j,i} I_{j,t} & \text{if } i \neq j \end{cases}$$

We produced summaries of this process both in terms of total cases contributed from one area to another and total cases normalized by epidemic size in each area as seen in Figure 7.

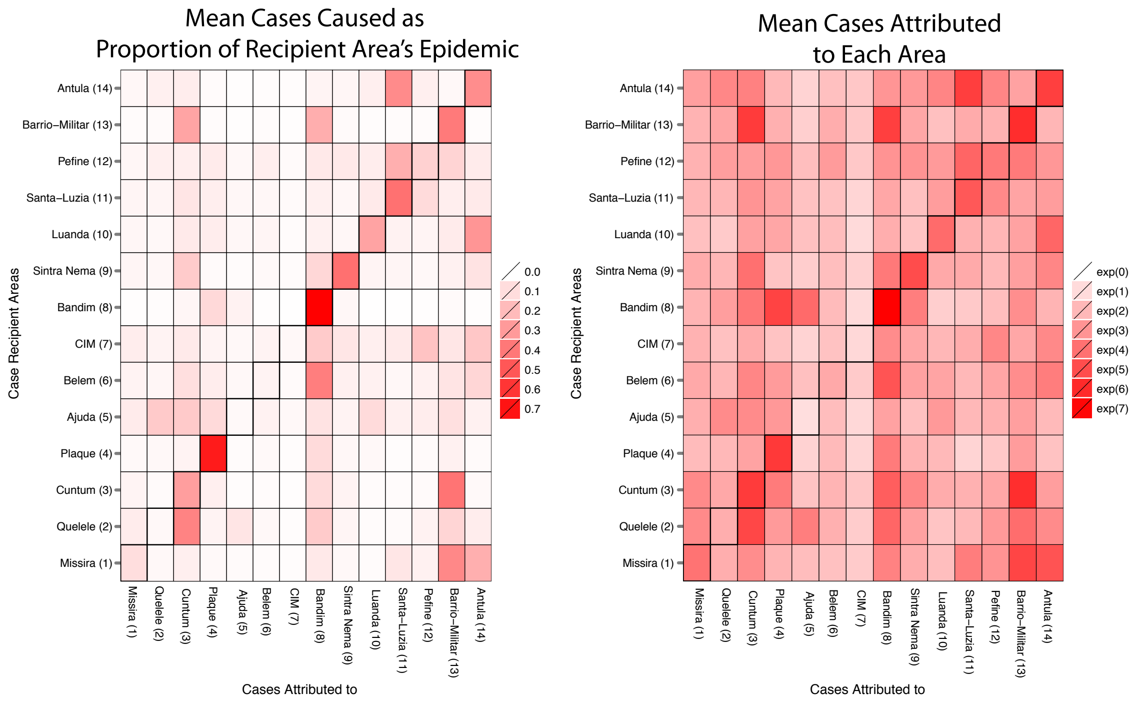

Figure 7: Mean cases attributed to different areas over the entire simulated epidemic. The right panel shows the proportion of each area's epidemic (rows) attributed to each area. The left panel shows the number of in each area attributed to others

References

[1] Plummer M (2003) JAGS: A Program for Analysis of Bayesian Graphical Models Using Gibbs Sampling. In: Proceedings of the 3rd International Workshop on Distributed Statistical Computing (DSC 2003). Vienna, Austria.

[2] Longini IM, Yunus M, Zaman K, Siddique A, Sack RB, et al. (2002) Epidemic and endemic cholera trends over a 33-year period in Bangladesh. Journal of Infectious Diseases 186: 246.

[3] Constantin de Magny G, Guégan JF, Petit M, Cazelles B (2007) Regional-scale climate-variability synchrony of cholera epidemics in West Africa. BMC Infectious Diseases 7: 20.

[4] Koelle K, Rodó X, Pascual M, Yunus M, Mostafa G (2005) Refractory periods and climate forcing in cholera dynamics. Nature 436: 696–700.

[5] Hastie T (2008) gam: Generalized additive models. R package version 1-06-1 .
